# Site-Specific Antigen Immobilization Improves Autoantibody Binding Efficiency on the Luminex Platform

**DOI:** 10.1021/acsomega.5c11987

**Published:** 2026-04-10

**Authors:** Dajana Kolanovic, Manuela Hofner, Jasmin Huber, Andreas Weinhaeusel, Birgit Wiltschi

**Affiliations:** † acib − Austrian Centre of Industrial Biotechnology, Petersgasse 14, 8010 Graz, Austria; ‡ Institute of Molecular Biotechnology, 27253 Graz University of Technology, Petersgasse 14, 8010 Graz, Austria; § Molecular Diagnostics, AIT Austrian Institute of Technology GmbH, Giefinggasse 4, 1210 Vienna, Austria; ∥ Institute of Bioprocess Science and Engineering, Department of Biotechnology and Food Sciences, BOKU University, Muthgasse 18, 1190 Vienna, Austria

## Abstract

Autoantibodies (AABs) are valuable biomarkers for diagnosing and monitoring autoimmune diseases and cancer. Conventional AAB profiling methods, such as enzyme-linked immunosorbent assay and immunoblot, are time-consuming, labor-intensive, and limited in multiplexing capacity. Luminex xMAP technology overcomes these limitations by enabling high-throughput, multiplexed AAB detection via bead-based immunoassays. However, the random immobilization of antigens on Luminex beads can lead to suboptimal epitope exposure, reduced binding sensitivity, and inconsistent assay performance. This study examines whether oriented antigen immobilization via genetic code expansion and click chemistry enhances binding sensitivity on the Luminex platform compared to random immobilization via conventional amine coupling. We selected three human antigenic proteins, HDAC3, RPS17, and RPS4Y1, and incorporated the noncanonical amino acid (ncAA) *N* ^ε^-((2-azidoethoxy)­carbonyl)-l-lysine (AzK) at a genetically defined position using the stop codon suppression method. This enabled site-specific conjugation of the antigens to dibenzocyclooctyne (DBCO)-functionalized beads via strain-promoted azide−alkyne cycloaddition (SPAAC). Binding sensitivity was assessed using serum samples from 88 individuals (22 healthy and 66 lung carcinoma patients). Oriented immobilization of RPS4Y1 AzK on DBCO-beads resulted in a 2.5-fold increase in binding sensitivity compared to random immobilization on COOH-beads, demonstrating that controlled antigen orientation improves epitope accessibility and enhances AAB detection sensitivity. These findings establish site-specific antigen immobilization via genetic code expansion and click chemistry as a superior alternative to conventional amine coupling. This immobilization approach significantly improves AAB detection and holds broad potential for applications such as antibody profiling, diagnostics, and drug screening on the Luminex and other biosensing and diagnostics platforms, where high sensitivity and accuracy are essential.

## Graphical abstract

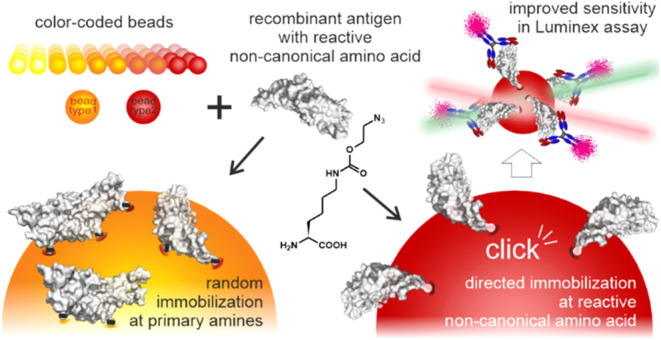

## 1 Introduction

Autoantibodies (AABs) are antibodies that target the body’s own proteins and are present in both healthy and diseased individuals. While the role of AABs in healthy individuals is not yet fully understood, they are believed to contribute to immune homeostasis, regulation of immune responses, infection resistance, and the modulation of biologically active molecules.^1^ However, when class-switched, high-affinity IgG AABs bind to endogenous antigens, they can trigger specific immune responses that may damage tissues or impair their function, potentially leading to autoimmune diseases.^2^ Additionally, AABs are frequently detected in cancer patients, where they develop in response to tumor-associated antigens.^3^ Given their association with various pathological conditions, AABs serve as valuable biomarkers for diagnosis, prognosis, and monitoring the progression of diseases.^4^ They are particularly useful for enabling early diagnosis, which is crucial for diseases that require timely therapeutic intervention.^5,6^ This asset has driven significant interest in developing fast, sensitive and high-throughput methods for AAB profiling.

Traditional techniques for AAB detection, such as enzyme-linked immunosorbent assay (ELISA), immunoblot, and immunoprecipitation, have proven impractical due to their time-consuming, labor-intensive nature, the necessity of large sample volumes, and elevated costs. Protein−antigen microarrays represent a more advanced alternative, where multiple antigens are immobilized on a functionally coated solid surface, such as glass slides.^7^ While microarrays offer key advantages including multiplexing capabilities, i.e., simultaneous detection of multiple analytes, and reduced sample volume requirements, they also have limitations. For instance, microarray production and processing are complex, requiring specialized equipment such as high-resolution microarray scanners and software for data extraction. Additional limitations include slow solid-phase kinetics, instability of immobilized antigens, and poor reproducibility.^8^

To address these challenges, Luminex introduced a more efficient multiplexing solution with its multianalyte profiling (xMAP) technology. Compared to microarrays, xMAP technology offers improved processing speed due to faster kinetics in solution-phase reactions. It also requires less sample volumes and fewer consumables, while allowing higher sample throughput and generating more data output with the same level of effort as planar microarray technologies.^9,10^ One additional advantage of the xMAP technology is its flexibility to support various assay formats, making it applicable to a wide range of analytical needs.^11^ When implemented as an immunoassay, xMAP technology primarily employs three assay formats: capture sandwich assays, competitive assays, and indirect (serological) assays.^9^ Both capture sandwich and competitive assays use immobilized antibodies (ABs) to capture target molecules, though they differ in their detection mechanisms. In contrast, the indirect serological assay, the format we used in the current study, utilizes an immobilized antigen to capture specific AABs from patient samples, which are then detected using an isotype- and species-specific labeled detection AB.

xMAP technology utilizes microspheres (beads) that can be either nonmagnetic or magnetic, with different surface chemistries tailored to specific applications. The most commonly used are MagPlex microspheres, Luminex’s latest generation of xMAP beads. These superparamagnetic beads are doped with three distinct fluorochromes (infrared, violet, and orange).^9,12^ Their magnetic properties allow for rapid and easy separation from the solution using a magnet, which simplifies washing steps and automation. While all MagPlex beads share the same fundamental properties, they differ in the amount of incorporated fluorochromes, generating a unique emission spectrum for each bead when excited by a red laser at 635 nm.^12^ This spectrum is detected by the xMAP instrument, allowing for precise differentiation and identification of each bead type in a multiplexed assay. By combining three distinct fluorescent dyes in varying concentrations, xMAP technology generates 500 uniquely color-coded bead types, each of which can be coated with a different ligand (antigen). Individual antigen-coupled bead types can be pooled into a single reaction well, enabling the simultaneous detection of up to 500 analytes, e.g., AABs binding to 500 different antigens within the same well. These binding events are detected using a secondary fluorescently labeled detection antibody, e.g., antihuman IgG. To facilitate detection, the xMAP instrument is equipped with a second light source, a green laser (532 nm). This laser excites the fluorochrome conjugated to the detection antibody and quantifies antigen-binding antibodies by assigning the measured median fluorescence intensity (MFI) to the corresponding identified bead type. By integrating signals from the red and green lasers, xMAP technology enables efficient processing and analysis of multiplexed assays.^9,10^ An overview of the xMAP technology workflow is illustrated in Figure 1.

**Figure 1:**
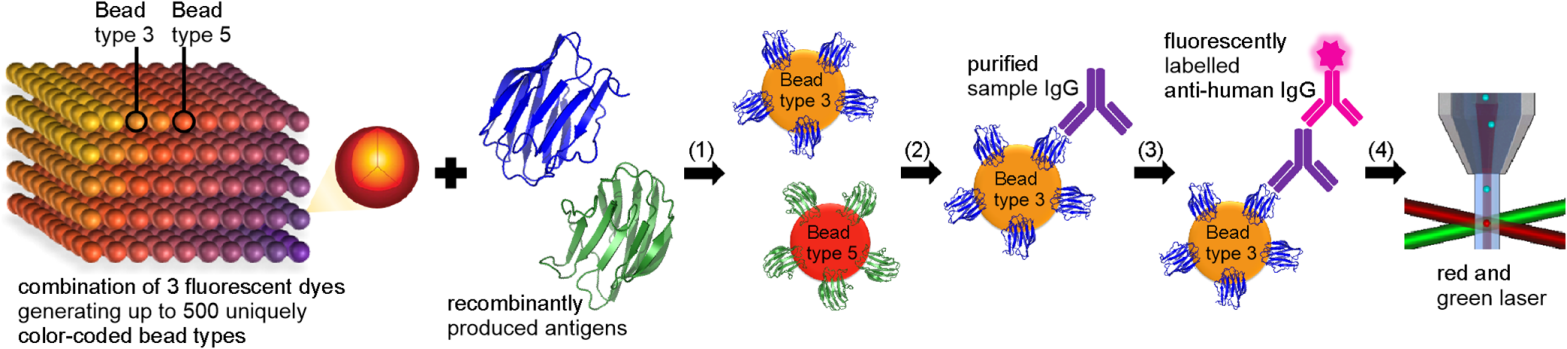
Schematic overview of the xMAP technology workflow. xMAP technology utilizes microspheres doped with three distinct fluorochromes in varying concentrations, generating up to 500 uniquely color-coded bead types. (1) Each bead type is coupled with a different antigen and pooled into a single reaction well. (2) Specific IgG autoantibodies (AABs) from patient samples bind to their corresponding antigen-immobilized bead types. (3) Bound AABs are detected using a fluorescently labeled secondary antibody, such as antihuman IgG. (4) Signal detection is performed by the xMAP instrument, which uses a red laser to identify each bead type and a green laser to quantify bound antibodies. By integrating signals from both lasers, xMAP enables efficient multiplexed analysis of up to 500 analytes per well.

The surface of MagPlex beads is functionalized with ∼10 ^8^ free carboxyl (COOH) groups, which enables the covalent attachment of capture molecules via amine coupling.^9^ This process forms a covalent amide bond between the bead’s carboxyl groups and the primary amines on the capture molecules. While amine coupling is a widely used and straightforward immobilization method, it is nonspecific when applied to proteins, e.g., antibodies or antigens, as it relies on the amino-terminus and the epsilon-amino group of lysine residues, which are abundant in proteins. Consequently, this method most often results in a random orientation of immobilized proteins, leading to hindered active site or epitope accessibility and nonuniform binding, ultimately compromising binding sensitivity and signal accuracy.^13,14^ To overcome these challenges, site-specific immobilization techniques have been developed. These methods enable controlled coupling of proteins to the surface, ensuring optimal orientation of their active sites or epitopes for interaction with the analyte, leading to improved binding sensitivity and reproducibility.^15–17^

To achieve site-specific protein immobilization, a unique functional group must be introduced at a defined position within the protein. Ideally, this functional group should be bioorthogonal, which means it does not naturally occur in proteins or other biomolecules and does not cross-react with endogenous functional groups. The presence of such a bioorthogonal functional group at a defined site in the protein enables its selective, site-specific and oriented coupling to a complementary reactive group on the solid surface. Bioorthogonal chemistries such as copper­(I)-catalyzed azide−alkyne cycloaddition (CuAAC) and strain-promoted azide−alkyne cycloaddition (SPAAC), commonly known as ‘click’ chemistries, have proven to be highly effective for protein modifications^18–20^ including directed immobilization.^21–24^ Given that azide and alkyne moieties do not naturally occur in recombinant proteins, a variety of strategies have been developed to introduce these functional groups in a site-specific manner. Chemical approaches typically rely on bifunctional linkers that react with canonical amino acid side chains, most commonly lysines or cysteines, but these methods often suffer from limited positional control and heterogeneity. Enzymatic and chemoenzymatic strategies offer an alternative route to site-specific modification, for example by exploiting native N-glycosylation sites on proteins. In such approaches, enzymes including endoglycosidases, glycosyltransferases, or transglutaminases can be used to remodel glycans or introduce bioorthogonal handles at defined locations, enabling subsequent click-based conjugation.^25–30^ While these methods can provide high specificity, their applicability depends on the presence and accessibility of suitable glycosylation or enzyme-recognition sites within the target protein. In contrast, genetic encoding of bioorthogonal functional groups provides direct and precise control over the incorporation site independent of native post-translational modifications. Noncanonical amino acids (ncAAs) bearing azide or alkyne side chains are not encoded by the standard genetic code but can be site-specifically incorporated into proteins during ribosomal translation under tightly controlled conditions. This is achieved using an in-frame amber stop codon, which is decoded by a suppressor tRNA_CUA_ charged with the reactive ncAA by an aminoacyl-tRNA synthetase (aaRS). While some wild-type aaRSs can recognize and charge the ncAA onto tRNA_CUA_, mutant aaRSs are often used due to their enhanced efficiency in charging ncAAs.^31,32^ Additionally, the aaRS/tRNA_CUA_ pair must be orthogonal in the host organism, which means that the aaRS does not charge any of the host’s native tRNAs with the ncAA, nor is the tRNA_CUA_ charged with a canonical amino acid by any of the host’s aaRSs.^33^ This method, known as ’stop codon suppression’ (SCS), allows the genetic encoding of a bioorthogonal group at a strategically chosen site in the gene of interest. Consequently, SCS facilitates the production of site-specifically functionalized proteins for controlled and oriented immobilization on surfaces.

Genetic code expansion technologies for ncAA incorporation are well-established and have found widespread applications in various fields, including protein immobilization.^34–37^ Several studies have compared ncAA-based immobilization with classic random immobilization methods. The results have demonstrated that oriented immobilization via ncAAs, combined with click chemistry, significantly enhances protein binding sensitivity.^38–40^ However, to the best of our knowledge, this approach has not yet been explored for antigen immobilization on the Luminex platform. Given the limited surface functionalities available on Luminex microspheres, capture molecules, e.g., antigens and antibodies, have mainly been immobilized in a random manner. Specifically, immobilization on the carboxylated MagPlex beads primarily rely on amine coupling, which results in nonspecific attachment and variable antigen orientations, potentially leading to reduced binding efficiency.^41^ To overcome these limitations, researchers have turned to genetic fusion strategies, incorporating specific binding tags such as glutathione-S-transferase (GST), SpyTag or biotin binding protein rhizavidin (RZ) to achieve oriented immobilization on the capture surface.^42–45^ Although this approach can be effective, it has several limitations. Genetic fusions typically restrict conjugation to either the N-terminus or C-terminus of proteins, which limits site-selective conjugation and prevents the selection of optimal attachment points. This restriction can hinder epitope accessibility and compromise binding sensitivity.^46–48^ Additionally, fusion tags may interfere with protein folding, activity, or expression levels, further impacting assay performance.^49,50^ Furthermore, using the biotin-binding protein in orientation strategies is incompatible with avidin−biotin detection systems, which are widely used for signal generation on the Luminex platform. To address the limitations of genetic fusions, we aimed to exploit genetic code expansion and click chemistry as an alternative approach for achieving oriented and controlled antigen immobilization on Luminex MagPlex beads. Specifically, we investigated whether oriented antigen immobilization via ncAA incorporation and click chemistry could enhance binding sensitivity compared to random amine coupling. As model antigens, we selected histone deacetylase 3 (HDAC3), ribosomal protein S17 (RPS17) and ribosomal protein S4 Y-linked 1 (RPS4Y1). To functionalize these human antigenic proteins with an azide-reactive handle for oriented immobilization, we employed the SCS method with the orthogonal pyrrolysyl-tRNA synthetase (PylRS)/tRNA_CUA_^Pyl^ pair from *Methanosarcina mazei* (*Mm*) in *E. coli*. This enabled the site-specific incorporation of the bioorthogonally reactive ncAA N^ε^-((2-azidoethoxy)­carbonyl)-l-lysine (AzK) at a single, genetically defined position in each antigen. The HDAC3, RPS17 and RPS4Y1 AzK variants were used for site-specific immobilization on DBCO-functionalized beads via SPAAC. However, the immobilization of these proteins was initially challenged by nonspecific binding, which we address in detail in this study. We successfully minimized nonspecific binding for RPS4Y1 AzK, achieving its oriented immobilization on DBCO-functionalized beads via SPAAC. For comparison, we randomly immobilized RPS4Y1 AzK on COOH-functionalized beads via amine coupling. To assess the impact of the immobilization strategy on binding sensitivity, we measured the binding signal intensities of oriented and randomly immobilized RPS4Y1 AzK using 88 patient serum samples (22 from healthy individuals and 66 from lung carcinoma patients). This study focuses solely on the technical aspects of immobilization efficiency, investigating whether oriented antigen immobilization improves epitope accessibility for AAB interactions, resulting in stronger signal detection compared to random immobilization. It does not address potential biological variables or broader clinical implications of this approach.

Our results demonstrate that oriented immobilization of RPS4Y1 AzK via ncAA incorporation and SPAAC significantly enhances binding efficiency toward serum AABs compared to random immobilization. These findings underscore the importance of antigen orientation in improving epitope accessibility and binding sensitivity. They also highlight the potential of genetic code expansion combined with click chemistry as powerful tools for improving assay performance on the Luminex platform, where high sensitivity, accuracy and reproducibility are essential.

## 2 Results and Discussion

### 2.1 Construction of Human Antigenic Proteins for Immobilization

To prepare the human antigenic proteins RPS17, RPS4Y1, and HDAC3 for directed immobilization, we referred to the constructs designed by Büssow.^51^ These constructs feature an N-terminal four amino acid linker (MRGS), followed by a hexahistidine (6H)-tag for protein purification and a linker-adapter sequence (GSYLGDTIESSTHAS) preceding the protein of interest. For amber mutants, we strategically inserted an amber codon at position 5 in the protein sequence, immediately upstream of the 6H-tag (Table S1). This design facilitates site-specific AzK incorporation for subsequent immobilization while eliminating any potential interference from truncated proteins in the further study. The 6H tag enables protein purification by immobilized metal affinity chromatography on a Ni^2+^-sepharose matrix (Ni-IMAC). Since the 6H-tag is expressed only when readthrough of the in-frame amber codon occurs, our design ensured that only full-length, AzK-containing protein variants were recovered for downstream experiments. Truncated proteins resulting from unsuppressed amber codons and premature translation termination lacked the 6H-tag necessary for Ni-IMAC purification and thus were not isolated by this method.

### 2.2 AzK Variants of Human Antigenic Proteins Were Successfully Produced

To achieve site-specific functionalization of human antigenic proteins with an azide-reactive handle for directed immobilization, we employed the SCS method using the orthogonal *Mm*PylRS/*Mm*tRNA_CUA_^Pyl^ pair. *Mm*PylRS specifically charges *Mm*tRNA_CUA_^Pyl^ with (pyrro)­lysine derivatives, including AzK. The charged tRNA_CUA_^Pyl^ then decodes the in-frame amber stop codon (UAG) on the mRNA and incorporates AzK into the growing polypeptide chain during translation.^32^ Synthetic gene fragments encoding RPS17, RPS4Y1, and HDAC3, each containing an amber codon (TAG) at position 5, were cloned into the pT7x31 expression vector, as detailed in the Materials and Methods section. In this vector, the expression of the target protein, *Mm*PylRS, and *Mm*tRNA_CUA_^Pyl^ are controlled by the T7 promoter,^52^ ensuring enhanced incorporation of the ncAA. The *E. coli* BL21­(DE3) expression strain was transformed with the constructs and cultivated overnight at 28 °C with vigorous shaking. To assess whether the location of the ncAA incorporation site influenced the expression of the RPS17, RPS4Y1, and HDAC3 amber mutants, we collected cell samples before and 18 h after AzK addition during IPTG induction (Figure 2A). The wild-type (wt) genes, *RPS17 wt*, *RPS4Y1 wt*, and *HDAC3 wt*, were expressed as benchmark controls (Figure 2B), and all collected samples were analyzed by SDS-PAGE. On the SDS-PA gel, we observed the protein bands at ∼24, 37, and 57 kDa only when IPTG and AzK were added, while the protein bands did not occur in the absence of IPTG and AzK (Figure 2A, lane 2 vs lane 1). This finding indicated that all three human protein AzK variants were successfully produced in *E. coli*. Furthermore, SDS-PAGE analysis revealed that SCS with AzK produced all three protein AzK variants without significant variation in their expression levels compared to their respective wild-type proteins (Figure 2, panel A vs panel B). However, expression levels varied among the three proteins. While RPS17 AzK and RPS4Y1 AzK exhibited similar expression levels, HDAC3 AzK showed the lowest (Figure 2A).

**Figure 2:**
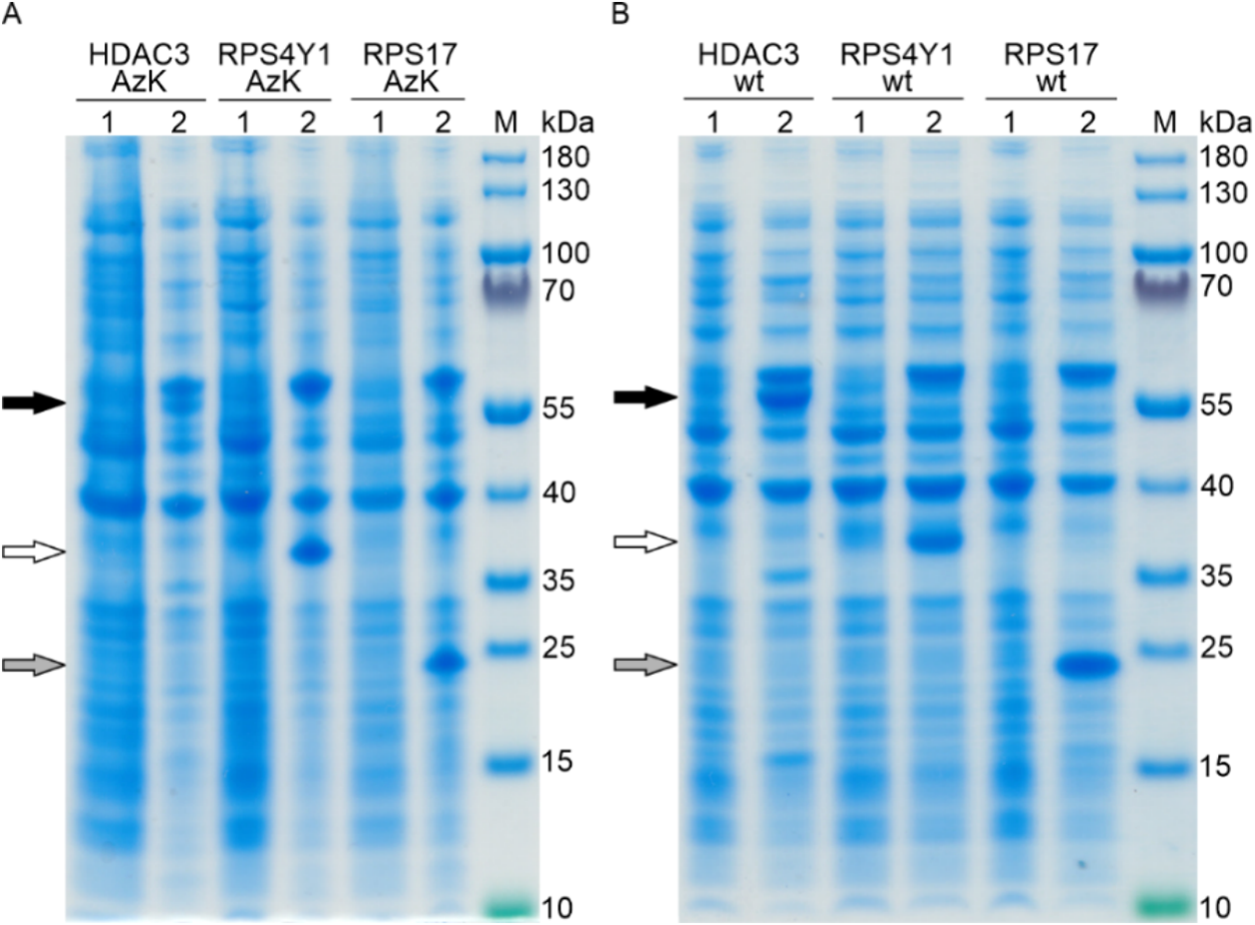
HDAC3, RPS4Y1, and RPS17 amber mutants were successfully expressed in the presence of AzK in *E. coli* BL21­(DE3). (A) Site-specific incorporation of AzK at position 5 in human antigenic proteins HDAC3, RPS4Y1, and RPS17. Whole cell samples were collected before (lane 1) and 18 h after AzK addition during IPTG induction (lane 2). (B) Wild-type genes, *HDAC3 wt*, *RPS4Y1 wt*, and *RPS17 wt*, were expressed as benchmark controls. Samples were collected before (lane 1) and 18 h after IPTG induction (lane 2). Lane M, molecular size marker. The numbers on the right margins of the gels represent the size of the molecular weight (MW) marker bands in kDa. Black arrows indicate HDAC3 wt (calculated molecular weight, MW_calc_ = 51.6 kDa) and HDAC3 AzK (MW_calc_ = 51.9 kDa), white arrows indicate RPS4Y1 wt (MW_calc_ = 32.2 kDa) and RPS4Y1 AzK (MW_calc_ = 32.5 kDa), and gray arrows indicate RPS17 wt (MW_calc_ = 18.3 kDa) and RPS17 AzK (MW_calc_ = 18.6 kDa). All proteins migrated at apparent MWs higher than their MW_calc_ on the Comassie-stained 4−12% Bis-Tris SDS-PA gel.

On the SDS-PA gel, the RPS17 wt, RPS4Y1 wt, and HDAC3 wt proteins migrated at apparent molecular weights of ∼24, 37, and 57 kDa, respectively, which were notably higher than their calculated molecular weights (MW_calc_) of ∼18, 32, and 52 kDa. The same migration pattern was observed for their AzK proteins (Figure 2A). Such aberrant protein migration behavior on SDS-PA gels has been widely reported and is often observed in proteins with a high proportion of acidic or basic amino acids.^53–56^ Since HDAC3 with a predicted isoelectric point (pI) of 5.2 is an acidic protein, the observed anomalous electrophoretic mobility is likely due to its high content of acidic amino acids. On the other hand, RPS17 and RPS4Y1, with pI values of 9.7 and 10.2, respectively, are highly basic proteins, and their aberrant migration can be attributed to their high content of basic amino acids. Nevertheless, we excised the overexpressed protein bands at ∼24, 37, and 57 kDa and unequivocally confirmed the identities of RPS17 wt, RPS4Y1 wt, and HDAC3 wt and their AzK variants by in-gel tryptic digestion and tandem mass spectrometry (Figure S1). Importantly, mass spectrometry analysis further confirmed the successful incorporation of AzK by identifying AzK-modified peptides in the RPS17, RPS4Y1, and HDAC3 azido variants.

To assess the solubility of AzK-functionalized proteins, we prepared soluble and insoluble protein fractions as described in the Materials and Methods section. SDS-PAGE analysis revealed that all three AzK protein variants were predominantly insoluble (Figure 3, lanes 3), which was consistent with the solubility profile of the wt proteins (data not shown). Nevertheless, we solubilized the inclusion bodies and purified the proteins via their 6H-tag by immobilized metal affinity chromatography on a Ni^2+^-sepharose matrix. After extensive optimization of the purification protocol, SDS-PAGE analysis revealed that all six proteins, HDAC3 wt, RPS4Y1 wt, RPS17 wt (Figure S2, panels A-C) and their AzK variants (Figure S2, panels D-F), were successfully purified by Ni-IMAC. However, HDAC3 was obtained at very low quantity (Figure S2, panels A and D, lanes E), which correlates with its poor expression visible in Figure 2. On the other hand, RPS4Y1 wt, RPS17 wt and their AzK variants were purified in sufficient quantities for further use, reflecting these proteins’ abundance in the insoluble fractions (Figure S2, lanes L in panels B, C, E and F). The purified antigens were stored in their respective elution buffers, which differed slightly in composition depending on the purification protocol used for each antigen, as described in the Materials and Methods section.

**Figure 3:**
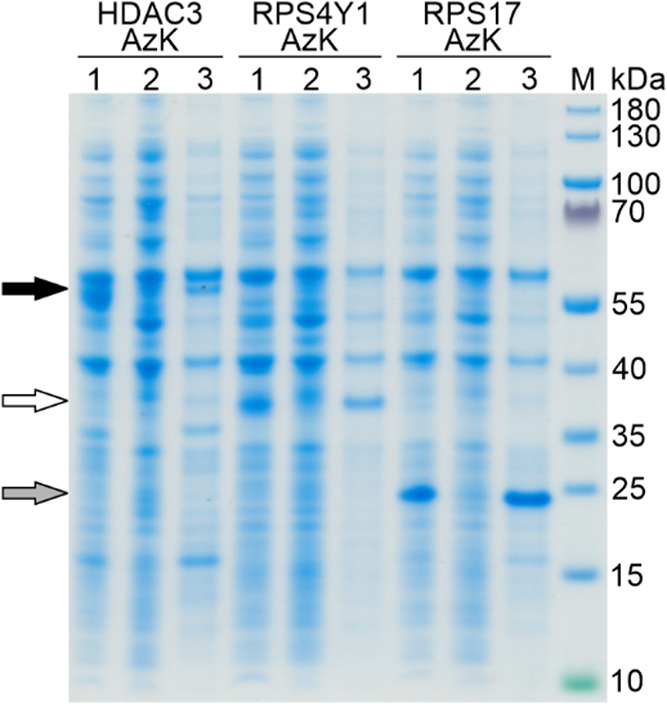
HDAC3 AzK, RPS4Y1 AzK, and RPS17 AzK variants were predominantly produced as insoluble proteins. Whole cell samples (lane 1), clarified lysates (lane 2) and solubilized inclusion bodies (lane 3) were prepared and subjected to SDS-PAGE. M, molecular size marker with sizes indicated in kDa on the right margin of the gel. The calculated molecular weights of HDAC3 AzK (black arrow), RPS4Y1 AzK (white arrow), and RPS17 AzK (gray arrow) are indicated in the caption to Figure 2.

### 2.3 Azide Groups in AzK-Functionalized Human Antigenic Proteins Remained Accessible for Conjugation

A critical requirement for downstream immobilization applications is that the genetically incorporated azido group at the selected position in AzK-functionalized protein variants remains chemically accessible for conjugation. To assess this, we conjugated IMAC-purified AzK-containing variants of RPS17, RPS4Y1, and HDAC3 with the fluorescent probe dibenzocyclooctyne-sulfo-Cyanine-3 (DBCO-Cy3) using strain-promoted azide−alkyne cycloaddition (SPAAC). SPAAC, also known as the “click” reaction, is a selective and highly efficient cycloaddition between a cyclic alkyne (e.g., DBCO) and an organic azide (e.g., AzK), forming a stable triazole product under mild conditions.^57^ Wild-type RPS17, RPS4Y1, and HDAC3 proteins, which lacked the reactive azide group for bioorthogonal conjugation, were used as negative controls to assess nonspecific probe binding. The reaction mixtures were analyzed by SDS-PAGE, followed by UV irradiation before Coomassie staining (Figure 4). The AzK-functionalized variants, RPS17 AzK, RPS4Y1 AzK, and HDAC3 AzK, exhibited fluorescent bands on the SDS-PA gel (Figure 4B, lanes 2, 4, 6), indicating successful conjugation of the azide groups with the alkyne-containing fluorophore. This finding confirmed that the incorporated azide groups in all three AzK variants were not only present but remained accessible for efficient bioorthogonal conjugation, an essential prerequisite for subsequent immobilization applications. In contrast, the RPS17, RPS4Y1, HDAC3 wild-type proteins, which lacked AzK, did not display a fluorescent band after treatment with DBCO-Cy3 (Figure 4B, lanes 1, 3, 5), confirming the absence of nonspecific fluorophore interaction.

**Figure 4:**
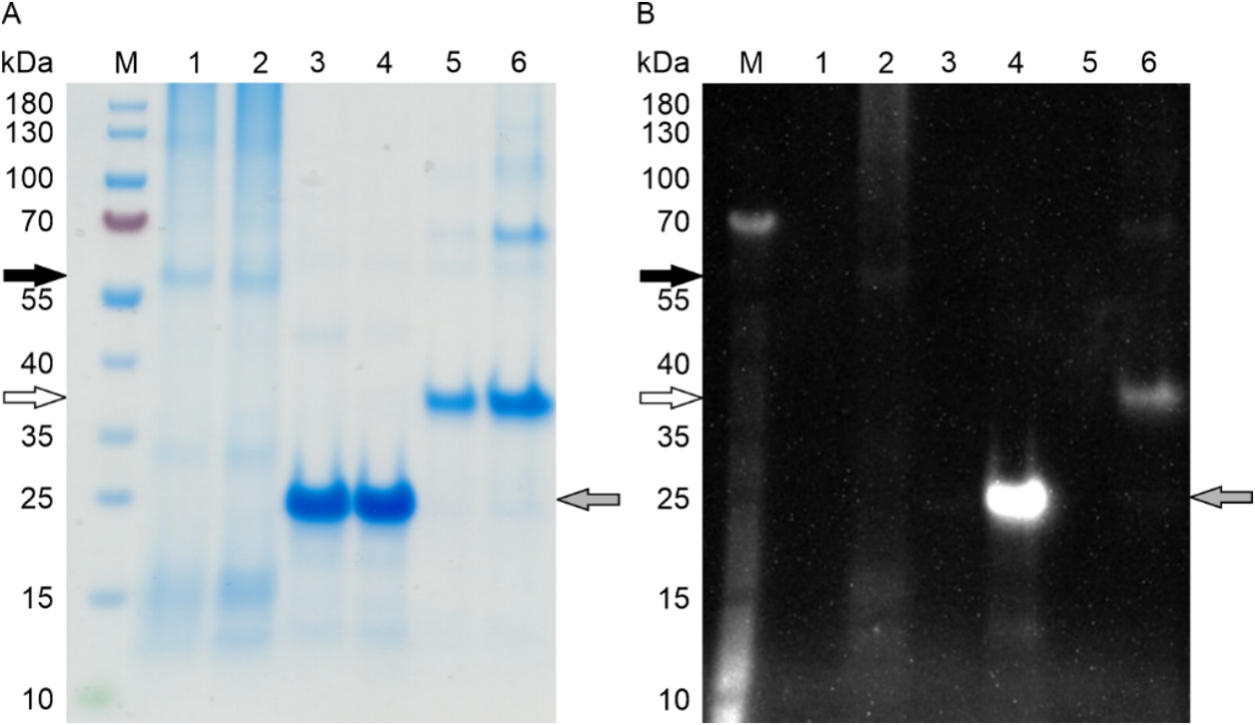
Fluorophore labeling via SPAAC confirmed accessibility of azide groups in AzK-functionalized human antigens. Following Ni-IMAC purification (Figure S2), IMAC-purified wild-type antigens and their AzK variants were incubated with the DBCO-Cy3 fluorophore and analyzed by SDS-PAGE. (A) Coomassie stain; (B) Exposure to UV light prior to Coomassie staining. Lane M, molecular size marker with sizes indicated in kDa on the left margins of the gels; lane 1, HDAC3 wt; lane 2, HDAC3 AzK; lane 3, RPS17 wt; lane 4, RPS17 AzK; lane 5, RPS4Y1 wt; lane 6, RPS4Y1 AzK. The calculated molecular weights of HDAC3 wt and HDAC3 AzK (black arrows), RPS4Y1 wt and RPS4Y1 AzK (white arrows), and RPS17 wt and RPS17 AzK (gray arrows) are provided in the caption of Figure 2.

Due to the initially low recovery of purified HDAC3 protein, which was insufficient for further experiments, we scaled up protein production to 1 L cultures. Following Ni-IMAC purification, SDS-PAGE analysis revealed improved expression levels, suggesting that the purified quantities of HDAC3 would be adequate for experiments on Luminex beads (data not shown). Additionally, successful conjugation of HDAC3 AzK with a fluorophore confirmed the presence and accessibility of the azido group in this protein variant (Figure S3).

### 2.4 AzK Antigen Variants Were Successfully Immobilized on DBCO-Agarose Beads

Knowing that azido labeled human proteins could be conjugated with small dye molecules, we next investigated whether they could also be immobilized on commercially available DBCO-agarose beads. This served as a preliminary step toward developing an immobilization protocol for Luminex beads. Due to the limited availability of HDAC3 AzK, we proceeded with RPS17 AzK and RPS4Y1 AzK for these preliminary experiments. Both AzK-functionalized proteins were incubated with DBCO-agarose beads for 24 h to allow conjugation via SPAAC. To assess nonspecific binding, wt RPS17 and RPS4Y1, which lacked AzK for bioorthogonal conjugation, were incubated under the same conditions with DBCO-agarose beads as negative controls. Additionally, wt and AzK protein variants were incubated without DBCO-agarose beads to assess protein stability under the conjugation conditions. Following the incubation, we analyzed the residual concentrations of RPS17 AzK and RPS4Y1 AzK in the reaction supernatants (Figure 5A, C). SDS-PAGE analysis revealed a decrease in their concentrations.

**Figure 5:**
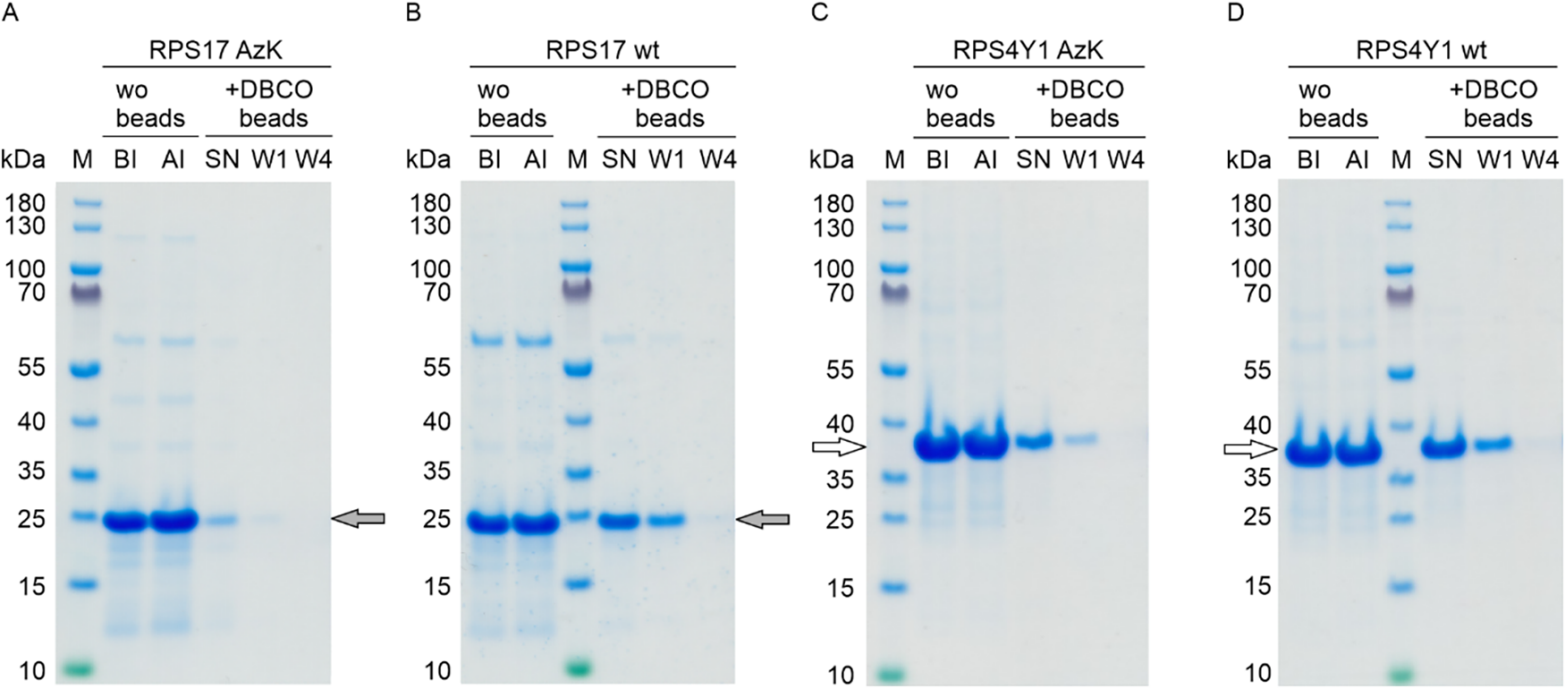
RPS17 AzK and RPS4Y1 AzK were successfully immobilized on the DBCO-agarose beads. SDS-PAGE analysis after incubation of (A) RPS17 AzK, (C) RPS4Y1 AzK, and their wt counterparts (B) RPS17 wt and (D) RPS4Y1 wt with or without DBCO-agarose beads. SN, supernatant after incubation with DBCO-agarose beads; W1 and W4, first and fourth wash fractions, respectively. Control antigen samples were incubated without beads (wo beads). Lane BI, before incubation; lane AI, after incubation. Lane M, molecular size marker with sizes indicated in kDa on the left margins of the gels. The calculated molecular weights of RPS17 wt and RPS17 AzK (gray arrows), and RPS4Y1 wt and RPS4Y1 AzK (white arrows) are provided in the caption to Figure 2.

To quantify the degree of immobilization of AzK-modified proteins, a mass balance analysis was performed using UV/vis spectrophotometric protein concentration measurements of the input material and the postincubation supernatant and wash fractions. Summing up the protein mass recovered in the supernatant and wash fractions and comparing it to the initial input, we determined that approximately 90% of RPS17 AzK and 81% of RPS4Y1 AzK were immobilized on the beads under these conditions. In contrast, the concentrations of RPS17 wt and RPS4Y1 wt in the supernatants remained unchanged, demonstrating that no nonspecific binding occurred on DBCO-agarose beads (Figure 5B,D). Furthermore, in the control samples without DBCO-agarose beads, RPS4Y1 AzK and RPS17 AzK remained in the supernatant, confirming that protein loss was not due to protein degradation or aggregation during the 24 h incubation period. These results demonstrate the successful and specific immobilization of RPS17 AzK and RPS4Y1 AzK on DBCO-agarose beads, paving the way for the development of immobilization protocols via ncAA and click chemistry for Luminex beads.

For RPS4Y1 AzK, a portion of the protein remained in the supernatant, suggesting incomplete immobilization. However, upon diluting the protein sample 8-fold, no detectable protein was present in the supernatant, indicating complete immobilization (Figure S4A). To further validate the immobilization, we performed an immunodetection assay using a rabbit anti-6H primary antibody followed by a horseradish peroxidase (HRP)-conjugated goat antirabbit secondary antibody, as described in the Materials and Methods section. Only the DBCO-agarose beads incubated with RPS4Y1 AzK developed a yellow color upon TMB substrate addition, confirming successful protein immobilization. In contrast, no yellow color was observed for beads incubated with RPS4Y1 wt, which demonstrated the specificity of the immobilization (Figure S4B). Absorbance measurements at 450 nm revealed a significantly higher signal of 1.8 for RPS4Y1 AzK-immobilized beads, compared to 0.6 for RPS4Y1 wt-incubated beads, further supporting the efficiency and specificity of the conjugation. Together, these results confirmed the successful and specific immobilization of RPS4Y1 AzK on DBCO-agarose beads.

### 2.5 Luminex Beads Were Successfully Functionalized with DBCO Groups for Oriented Antigen Immobilization

The use of AzK-functionalized antigens for immobilization on MagPlex beads requires additional bead surface modification with a linker molecule to introduce the appropriate reactive group i.e., DBCO groups for the SPAAC reaction (Figure 6, left). To functionalize the carboxylated (COOH) polystyrene MagPlex beads with DBCO groups, we employed a sulfo DBCO-PEG4-amine linker and performed amine coupling as described in the Materials and Methods section. Upon activation of the COOH groups, amine-reactive intermediates (sulfo-NHS esters) formed on the bead surface, facilitating the coupling of the sulfo DBCO-PEG4-amine linker. To prevent cross-reactivity of residual sulfo-NHS esters with protein amines during antigen immobilization, we performed an ethanolamine passivation step immediately after linker coupling. Ethanolamine was included at a concentration sufficiently high to quench any remaining reactive sulfo-NHS esters on the bead surface, thus eliminating accidental amine coupling during antigen immobilization. The successful modification of the beads was verified using an azide-PEG3-biotin conjugate, followed by a SPAAC reaction. The SPAAC reaction between the azide group of azide-PEG3-biotin and the alkyne group of the DBCO moiety conjugated biotin to the DBCO-functionalized beads. The biotin moiety enabled subsequent binding of Streptavidin-R-Phycoerythrin (SA-PE) for fluorescence-based detection. The resulting fluorescence signal, measured as median fluorescence intensity (MFI), confirmed the successful functionalization of beads with DBCO groups.

**Figure 6:**
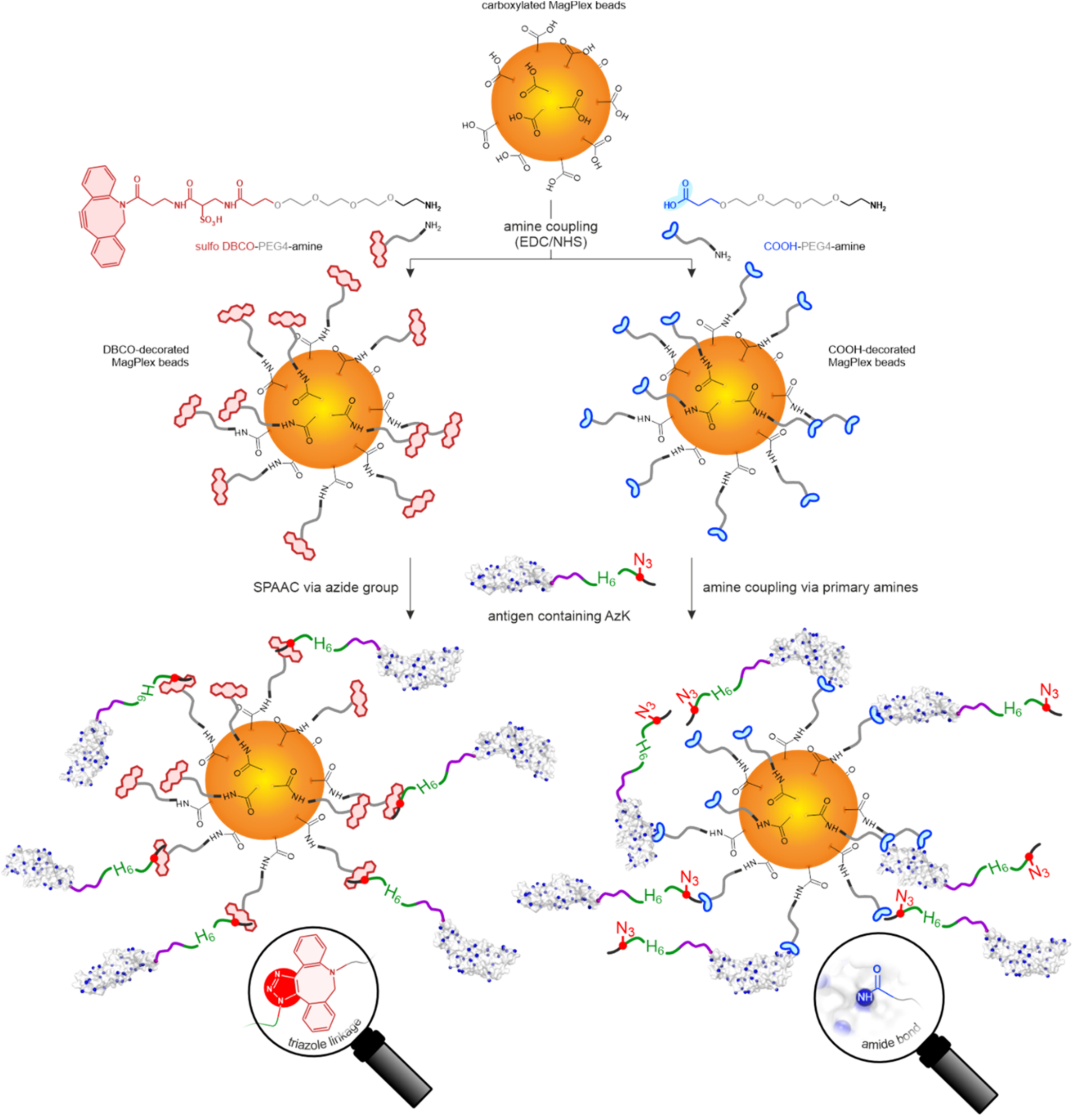
Schematic representation of the directed and random immobilization strategies used in this study. Carboxylated MagPlex beads were decorated with sulfo DBCO-PEG4-amine or COOH-PEG4-amine by amine coupling. AzK-labeled antigen proteins were immobilized either by SPAAC via the azido group (N_3_; directed immobilization, left) or by amine coupling via the primary amines (blue spheres; random immobilization, right). The colors of the N-terminal sequence, hexahistidine-tag (H6) and linker-adapter (purple) of the antigens correspond to the colors of the sequences listed in Table S1.

To ensure complete surface saturation with DBCO groups, we optimized the coupling process by testing various concentrations of the sulfo DBCO-PEG4-amine linker (Figure S5). The results demonstrated that a linker concentration of 250 μg/mL was sufficient for full conversion of the COOH groups into DBCO groups. To confirm the specificity of the functionalization process, we included a negative control in which the sulfo DBCO-PEG4-amine linker was added without prior EDC/sulfo-NHS activation. This resulted in no increase in the MFI, which confirmed the absence of nonspecific binding of the sulfo DBCO-PEG4-amine linker to the beads (Figure 7). Further, to verify the specificity of the functionalization process, we performed a second negative control in which EDC/sulfo-NHS activation was carried out, but the sulfo DBCO-PEG4-amine linker was omitted. This control also showed no increase in fluorescence, which indicated that azide-PEG3-biotin binding was specific and occurred only when the sulfo DBCO-PEG4-amine linker was successfully coupled to the beads (Figure 7). These findings verified that carboxylated MagPlex beads were effectively and specifically functionalized with DBCO groups, making them suitable for SPAAC-based protein immobilization.

**Figure 7:**
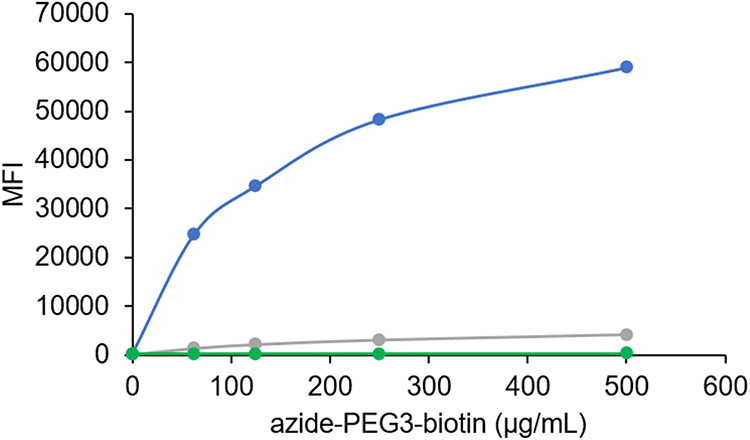
Functionalization of Luminex beads with DBCO groups was effective and specific. Carboxylated MagPlex beads were functionalized with DBCO groups via amine coupling using a sulfo DBCO-PEG4-amine linker at varying concentrations, as shown in Figure S5. Saturation of DBCO groups on the bead surface was achieved at a linker concentration of 250 μg/mL (dark blue line). Functionalization was verified via SPAAC with azide-PEG3-biotin at final concentrations of 500, 250, 125, and 62.5 μg/mL, allowing Streptavidin-R-Phycoerythrin (SA-PE) binding and fluorescence detection, measured as median fluorescence intensity (MFI). A control (blank) consisting of MES buffer without azide-PEG3-biotin (0 μg/mL) exhibited no fluorescence. To confirm the specificity of functionalization, two controls were included: beads incubated with sulfo DBCO-PEG4-amine linker without prior EDC/sulfo-NHS activation (light gray line), and beads subjected to EDC/sulfo-NHS activation without addition of the sulfo DBCO-PEG4-amine linker (light green line). Each azide-PEG3-biotin concentration, including the MES buffer control, was tested in duplicate. Data points represent the average MFI of two technical replicates.

To compare the performance of oriented antigen immobilization via SPAAC with random immobilization via amine coupling, we aimed to establish a direct side-by-side comparison. MagPlex beads are inherently functionalized with COOH groups, allowing random protein immobilization through amine coupling.^9^ To ensure a valid comparison between the two immobilization strategies, it was essential to establish a consistent structural architecture on the beads. For this purpose, we modified the COOH groups on MagPlex beads with an amino-PEG4-COOH linker via amine coupling (Figure 6, right), as described in the Materials and Methods section. This linker incorporated a PEG4 spacer, identical to that used in the DBCO-functionalized beads, ensuring consistency in spacer length and structural properties between the beads for both immobilization strategies. As with DBCO-functionalization, ethanolamine passivation was performed immediately after coupling the amino-PEG4-COOH linker to prevent unwanted cross-reactivity. Successful modification of the beads with COOH groups was validated using an amine-PEG3-biotin conjugate. Amine coupling between the amine group of amine-PEG3-biotin and the COOH group of the amino-PEG4-COOH linker facilitated biotin conjugation to the COOH-functionalized beads, which enabled subsequent SA-PE binding for fluorescence detection.

To optimize conversion efficiency, we tested different concentrations of amino-PEG4-COOH linker (Figure S6A). The highest concentration tested (100 μg/mL) was equimolar to the 250 μg/mL of sulfo DBCO-PEG4-amine, which had achieved saturation in the DBCO functionalization experiment. However, despite using the same molar ratio of linker to COOH groups as in the DBCO conversion, 100 μg/mL amino-PEG4-COOH did not achieve complete conversion (Figure S6A). Increasing the amino-PEG4-COOH linker concentration to 250 μg/mL, which matched the mass concentration used for sulfo DBCO-PEG4-amine, successfully drove the conversion into saturation (Figure S6B).

To validate the specificity of the functionalization process, we performed a control experiment in which amino-PEG4-COOH and amine-PEG3-biotin were added without prior EDC/sulfo-NHS activation of COOH groups. This resulted in no increase in MFI values, which confirmed that nonspecific binding of the amino-PEG4-COOH linker and amine-PEG3-biotin did not occur (Figure S6A). Additionally, to assess the efficacy of the ethanolamine passivation step, we performed another control. In this control, COOH groups on beads were activated with EDC/sulfo-NHS, but the amino-PEG4-COOH linker was not added. Following ethanolamine passivation, amine-PEG3-biotin was introduced without further EDC/sulfo-NHS activation. No increase in fluorescence was observed, confirming that ethanolamine effectively quenched amine-reactive intermediates (sulfo-NHS esters), thereby preventing unintended reactions with amine-PEG3-biotin (Figure S6A). Together, these results demonstrate that the passivation step was effective and that the amino-PEG4-COOH linker was specifically and successfully coupled to the Luminex MagPlex beads. The functionalization of carboxylated MagPlex beads with the amino-PEG4-COOH linker resulted in beads with an extended PEG4 spacer while retaining carboxyl functionality for subsequent amine coupling. To distinguish these beads from the original carboxylated MagPlex beads, they will hereafter be referred to as COOH-functionalized beads.

### 2.6 Immobilization of Human Antigenic Proteins on DBCO-Functionalized Beads Demonstrated Nonspecific Binding

After successfully converting carboxylated MagPlex beads into DBCO- and COOH-functionalized beads, our next objective was to immobilize AzK-labeled antigens and compare the performance of oriented versus random antigen immobilization (Figure 6). Specifically, we aimed to determine whether directed, single-site antigen immobilization at AzK enhances autoantibody binding sensitivity compared to random, multisite immobilization at amino groups.

For this purpose, we conjugated the azido-functionalized antigens HDAC3 AzK, RPS4Y1 AzK, and RPS17 AzK onto DBCO-functionalized beads via SPAAC. Wild-type (wt) antigens HDAC3, RPS4Y1, and RPS17, which lack the azido reactive group required for bioorthogonal conjugation, were included as negative controls to assess potential nonspecific binding. Antigen immobilization was validated using an anti-6H-tag-biotin antibody, followed by SA-PE binding, as detailed in the Materials and Methods section. The results confirmed that AzK-functionalized antigens were successfully immobilized on DBCO-functionalized beads (Figure S7, blue lines). Unexpectedly, wt antigens also bound to the DBCO-functionalized beads, despite the absence of the azido group required for SPAAC. This finding hinted at nonspecific interactions between the beads and antigens (Figure S7, orange lines).

To tackle the source of this nonspecific binding, we first considered hydrophobic interactions. Notably, the antigens used in this study were expressed in *E. coli* as insoluble inclusion bodies, a form often enriched in exposed hydrophobic regions due to improper protein folding. These hydrophobic patches, containing amino acids such as phenylalanine, leucine, and valine, can readily interact with hydrophobic surfaces.^58^ Since DBCO groups are inherently hydrophobic, they may promote nonspecific interactions with these exposed hydrophobic protein regions, even in the absence of the reactive azido group. To determine whether the observed nonspecific binding of wt antigens to DBCO-functionalized beads was indeed driven by hydrophobic interactions between the DBCO groups and the antigens, we evaluated their interaction with COOH-functionalized beads. These beads are identical to DBCO-functionalized beads except for the functional group on their surface. We incubated wt antigens with COOH-functionalized beads in the absence of EDC/sulfo-NHS reagents, which are required to activate the COOH groups for amine coupling. As a positive control, wt antigens were incubated with COOH-functionalized beads in the presence of EDC/sulfo-NHS. Remarkably, the results revealed that all three wt antigens, HDAC3 wt, RPS4Y1 wt, and RPS17 wt, bound to COOH-functionalized beads, regardless of EDC/sulfo-NHS activation, indicating that their binding resulted from nonspecific interactions (Figure S8). Given that the only difference between DBCO- and COOH-functionalized beads is their surface functional group, these findings confirmed that the nonspecific binding of wt antigens to DBCO-functionalized beads was not driven by the DBCO moiety itself but rather by the intrinsic properties of the bead matrix. MagPlex beads are primarily composed of polystyrene, a chemically inert yet highly hydrophobic material.^59^ The surface of polystyrene beads may contain regions that are not fully covered by functional groups, leaving exposed hydrophobic areas that can interact with hydrophobic protein regions. Our findings suggest that antigens may adsorb directly onto the polystyrene matrix via hydrophobic interactions, bypassing surface functional groups like DBCO and COOH, which leads to their nonspecific and nondirectional binding. To further emphasize the role of bead matrix composition in nonspecific interactions, it is worth noting that the RPS17 wt and RPS4Y1 wt antigens did not exhibit similar behavior when conjugated to DBCO-agarose beads. Unlike polystyrene, agarose is a hydrophilic material that does not promote hydrophobic interactions.^60^ Remarkably, neither RPS17 wt nor RPS4Y1 wt exhibited detectable binding to DBCO-agarose beads, despite the presence of DBCO groups (Figure 5). This finding further supports the hypothesis that the hydrophobic nature of the polystyrene matrix plays a major role in promoting nonspecific antigen binding.

In addition to hydrophobic interactions, we evaluated whether electrostatic interactions contributed to the observed nonspecific binding. Proteins often possess localized charges from arginine, lysine, aspartate, and glutamate residues,^61^ while the bead surface may carry charged groups depending on its composition and functionalization. Although optimized functionalization conditions confirmed that carboxyl groups on MagPlex beads were fully converted to DBCO groups (Figure S5), it is possible that some COOH groups remained unreacted due to steric hindrance. If present, these residual COOH groups would retain their negative charge at the pH of the coupling buffer (MES, pH 5). Since the pI values of HDAC3, RPS4Y1, and RPS17 are 5.2, 10.1, and 9.7, respectively, these antigens are positively charged during the coupling procedure (MES, pH 5) and could therefore interact electrostatically with any residual negatively charged COOH groups on the bead surface. To assess the potential contribution of electrostatic interactions, we increased the ionic strength of the washing buffer by adding different concentrations of NaCl, ranging from 300 mM to 1 M. Next, we adjusted the pH of the coupling buffer (e.g., PBS, pH 7.4) to bring it closer to the protein’s pI values, minimizing their net charge and consequently reducing electrostatic interactions. However, neither of these strategies significantly reduced nonspecific binding (data not shown), suggesting that electrostatic interactions are not the primary driver of the observed nonspecific binding. Since neither MES, pH 5 nor PBS, pH 7.4 coupling buffers reduced nonspecific binding, we reasoned that the buffer composition had minimal impact on this issue. Therefore, to streamline the workflow and maintain protein stability, we performed all subsequent coupling reactions directly in the antigens’ urea-containing elution buffers.

These findings further support the conclusion that hydrophobic interactions between the polystyrene bead matrix and hydrophobic regions of antigens are the dominant mechanism underlying nonspecific binding in our system. To address this, we focused on mitigating hydrophobic interactions, either by disrupting them through stringent washing conditions or by masking hydrophobic bead surfaces using hydrophilic blocking molecules.

### 2.7 Blocking of DBCO-Functionalized Beads Reduced Nonspecific Binding of RPS4Y1 Antigen

One approach to mitigate protein interactions with hydrophobic surfaces involves adjusting experimental conditions. Since detergents can disrupt hydrophobic interactions between proteins and surfaces,^62,63^ we tested the inclusion of the nonionic detergent Tween 20 (0.05−2% v/v) in the washing buffer and increased the number of washes from three to six. However, neither increasing the detergent concentration nor the number of washes significantly reduced nonspecific antigen binding to the DBCO-functionalized beads (data not shown). This finding indicates that antigen interactions with the polystyrene bead surface are particularly strong and resistant to disruption by detergent-based approaches. In addition, we observed that decreasing the antigen concentration in the coupling reaction did not significantly reduce nonspecific binding (data not shown), suggesting that the strength of antigen-polystyrene interactions is independent of antigen concentration. These findings highlight the need for alternative strategies to effectively minimize nonspecific binding.

Another strategy to reduce hydrophobic interactions involves modifying the bead surface properties, specifically by applying hydrophilic coatings to mask the inherent hydrophobicity of polystyrene. Hydrophilic blocking agents, such as bovine serum albumin (BSA) can occupy hydrophobic sites on the surface, and form a relatively stable, uniform layer that creates a steric and hydrophilic barrier against nonspecific protein interactions.^64,65^ To assess the efficacy of this strategy, we treated DBCO-functionalized beads with various blocking agents before adding the wt antigens. Seven different blocking agents were tested while PBS (which lacks blocking agents) served as a baseline control. The results showed that for RPS4Y1 wt, all tested blocking agents significantly reduced nonspecific binding compared to the PBS control (Figure 8A). However, for HDAC3 wt and RPS17 wt, none of the blocking agents reduced nonspecific binding to the DBCO-functionalized beads (Figure S9). The results also revealed variability in blocking efficiency across different blocking agents, particularly for RPS4Y1 wt. Notably, 1% and 0.05% (w/v) casein were the most effective in minimizing nonspecific binding of RPS4Y1 wt, which was in accordance with previous studies demonstrating the casein’s effectiveness in blocking polystyrene surfaces.^66–68^

**Figure 8:**
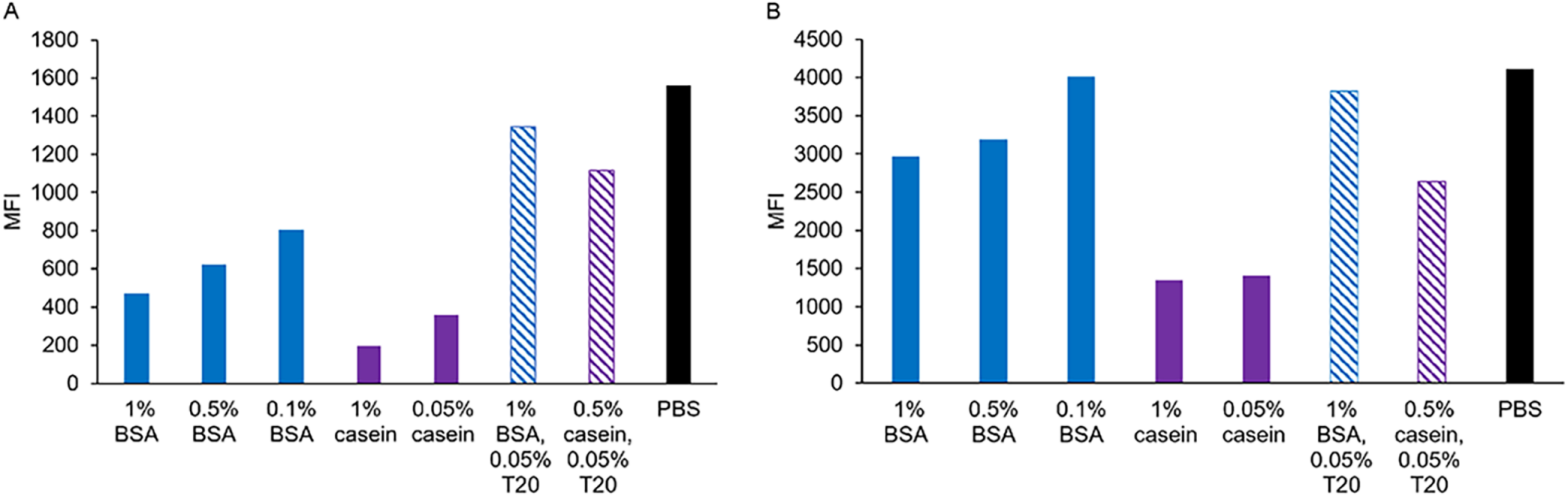
Blocking reduces nonspecific binding of RPS4Y1 antigen to DBCO-functionalized beads. To minimize nonspecific antigen adsorption, various concentrations of bovine serum albumin (BSA) (blue bars) and casein (purple bars) in PBS, pH 7.4 were tested, along with combinations containing 0.05% (v/v) Tween 20 (T20) (hatched bars). PBS alone served as a control (black bars). After incubation, antigen binding was detected using an anti-6H-tag-PE antibody at a final concentration of 10 μg/mL. Antibody detection was performed in duplicate and bars represent the average MFI of two technical replicates. (A) RPS4Y1 wt; (B) RPS4Y1 AzK. A control experiment confirming the absence of nonspecific interactions between blocking and detection reagents is shown in Figure S10.

Overblocking is another critical factor to consider, as excessive blocking can hinder specific protein binding. To evaluate this, we tested the coupling efficiency of RPS4Y1 AzK after blocking with each agent and PBS (Figure 8B). While 1 and 0.05% casein were the most effective at reducing nonspecific binding of RPS4Y1 wt, they also significantly impaired the specific binding of RPS4Y1 AzK. Among the tested agents, 1% BSA provided the optimal balance, significantly reducing nonspecific binding of RPS4Y1 wt without substantially compromising the specific binding of RPS4Y1 AzK. Therefore, 1% BSA was identified as the optimal blocking agent for the specific immobilization of RPS4Y1 AzK on DBCO-functionalized beads.

In the previous experiments, we used anti-6H-tag-biotin and SA-PE for detection. However, to minimize the risk of nonspecific binding of SA or biotin to the protein-based blocking agents, we switched to a directly conjugated antibody, anti-6H-tag-PE, in this experiment. To confirm that the observed fluorescence after blocking resulted from antigen interactions with the anti-6H-tag-PE detection antibody, we measured fluorescence from blocked DBCO-beads in the absence of antigen (Figure S10). As a reference, we incubated beads with PBS instead of blocking agent, representing unblocked beads. The results showed consistently low fluorescence signals (MFI ∼200) across all conditions. This confirmed that fluorescence detected after blocking originates from specific antigen interactions with the anti-6H-tag-PE rather than nonspecific binding of the detection antibody to blocking reagents.

The variable effectiveness of blocking with 1% (w/v) BSA across HDAC3 wt, RPS4Y1 wt, and RPS17 wt likely reflects intrinsic differences in protein properties, particularly their behavior under solubilizing and denaturing conditions. All three antigens were produced in *E. coli* as inclusion bodies and solubilized in 6 M urea at pH 8. Urea-mediated solubilization disrupts noncovalent interactions within protein aggregates, resulting in unfolded or partially unfolded species with increased exposure of hydrophobic residues.^69^ While urea partially shields these regions, the extent of hydrophobic surface exposure depends on protein-specific factors such as amino acid composition, folding propensity, and aggregate stability. In addition, urea solubilization efficiency is pH-dependent and can vary between proteins.^70^ Since all three antigens were solubilized at the same pH 8 condition, differences in solubilization efficiency likely resulted in varying degrees of exposed hydrophobic residues, contributing to protein-dependent nonspecific interactions with hydrophobic bead surfaces.

RPS4Y1 wt likely solubilized more efficiently at pH 8 than RPS17 wt and HDAC3 wt. This could have resulted in a more uniform denatured state with fewer persistent hydrophobic patches in comparison to the other two antigens. As a result, the reduced hydrophobic exposure could have allowed 1% BSA blocking to effectively mask hydrophobic sites on the bead surface, thereby minimizing nonspecific binding of RPS4Y1 wt. In contrast, RPS17 wt and HDAC3 wt might have been less efficiently solubilized at pH 8, retaining partially aggregated forms with a higher density of exposed hydrophobic surfaces. These persistent hydrophobic patches likely promoted stronger nonspecific interactions with the bead surface. Such strong interactions may overcome the steric and hydrophilic barrier imposed by the BSA layer, making blocking insufficient to prevent nonspecific binding.

Notably, this interpretation is supported by hydropathy analysis using the Kyte-Doolittle scale, which assesses the local hydrophobicity or hydrophilicity along a protein sequence.^71^ In this scale, positive values indicate hydrophobic regions, with scores above +1.5 typically marking strongly hydrophobic stretches, while negative values denote hydrophilic regions. The hydropathy plot of HDAC3 wt reveals extended, continuous hydrophobic stretches, with scores nearing or exceeding +2, which suggests a propensity for incomplete solubilization and nonspecific binding (Figure S11A). RPS17 wt shows a predominantly hydrophilic profile, but the presence of several peaks above +1 indicates hydrophobic patches that could mediate nonspecific interactions when not fully shielded (Figure S11B). In contrast, RPS4Y1 wt exhibits a more balanced hydropathy profile with alternating hydrophobic and hydrophilic regions and fewer extended hydrophobic stretches (only a few regions exceeded +2) (Figure S11C). This distribution likely supports more effective solubilization and reduced nonspecific interactions.

Based on these findings, subsequent immobilization experiments focused on RPS4Y1 AzK, as this antigen demonstrated the most favorable response to the blocking strategy showing reduced nonspecific interaction while maintaining specific binding.

### 2.8 Oriented RPS4Y1 AzK Antigen Immobilization Enhanced Autoantibody Binding Sensitivity

To evaluate whether oriented, single-site antigen immobilization at AzK improves AAB binding efficiency compared to random multisite immobilization at primary amines, we immobilized IMAC-purified RPS4Y1 AzK onto DBCO- and COOH-functionalized beads. The RPS4Y1 AzK antigen, engineered with a single azide group at a genetically predefined position, was conjugated to DBCO-beads via SPAAC, which enabled controlled and site-specific attachment. This precise conjugation strategy ensures uniform antigen orientation, thereby maximizing epitope accessibility and enhancing AAB binding efficiency. In contrast, RPS4Y1 AzK contains 21 primary amines, including 20 lysine residues and the N-terminus (Table S1; Figure S12), which serve as potential reactive sites for amine coupling with COOH-beads. Random conjugation at an unpredictable number and location of attachment sites leads to varying antigen orientations that may obstruct epitope accessibility and reduce AAB binding efficiency.

To assess the effect of immobilization strategy on binding sensitivity, we measured binding signal intensities of oriented and randomly immobilized RPS4Y1 AzK using serum samples from 88 patients. The cohort included 22 healthy individuals and 66 lung carcinoma patients (22 benign, 22 adenocarcinoma, and 22 squamous cell carcinoma). For the immobilization step, we loaded equal amounts of RPS4Y1 AzK, derived from the same purified protein sample (Figure S2E; 10E) onto DBCO- and COOH-beads. Successful coupling was confirmed by MFI of 59383 and 62805, respectively (Figure S13). The comparable coupling levels indicated that similar amounts of antigen were immobilized. Therefore, any differences in AAB binding could be attributed to the immobilization strategy i.e., epitope presentation on the bead surface rather than variations in antigen quantity. To account for background signals, we prepared negative controls by incubating DBCO- and COOH-beads with buffer alone instead of antigen. Plotting raw data as a line graph revealed that signal intensities were consistently higher across all tested samples when RPS4Y1 AzK was immobilized in an oriented manner on DBCO-beads compared to its random immobilization on COOH-beads (Figure 9A, orange vs dark blue line). Beads incubated with buffer alone produced significantly lower signals compared to those with immobilized protein (Figure 9A), indicating that nonspecific interactions between AABs and bead surfaces were minimal. To ensure accurate comparisons, background signals were subtracted from each data set before statistical analysis to isolate specific RPS4Y1 AzK-AAB interactions (Figure 9B). The normalized data further confirmed that oriented antigen immobilization improved binding sensitivity compared to random immobilization.

**Figure 9:**
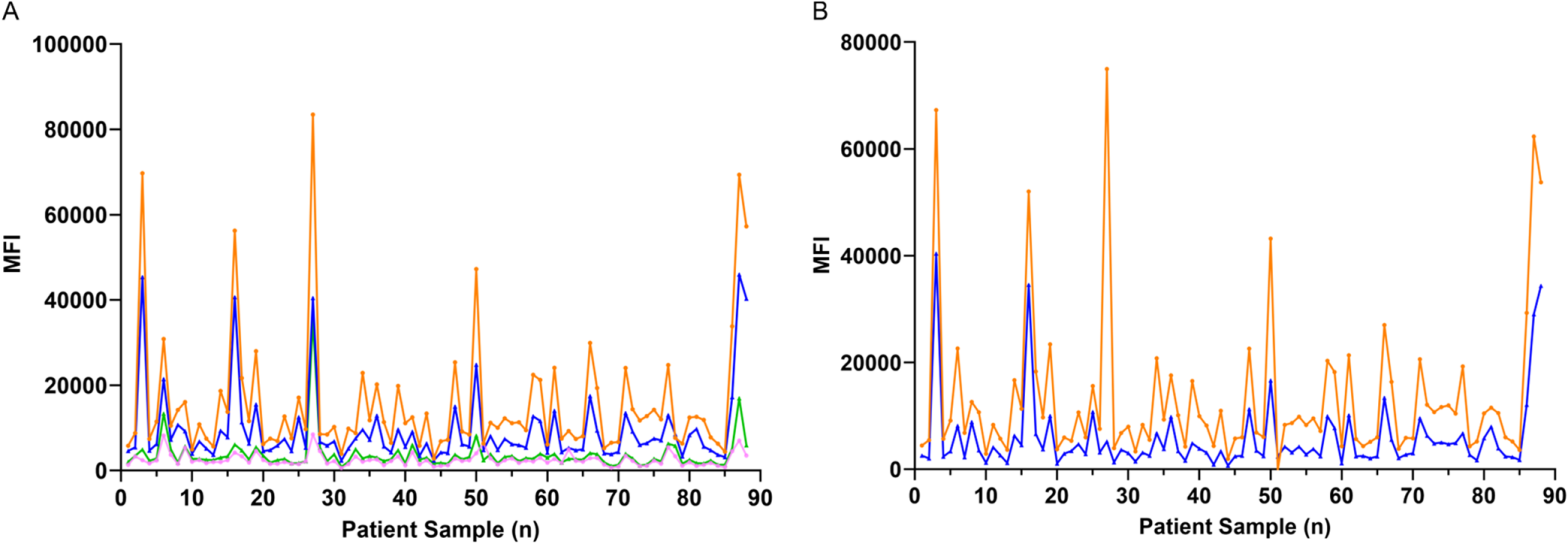
Oriented immobilization of RPS4Y1 AzK on DBCO-beads enhances AAB detection sensitivity compared to random immobilization on COOH-beads. To compare binding signal intensities between oriented and random antigen immobilization, IMAC-purified RPS4Y1 AzK was immobilized on DBCO- and COOH-functionalized beads and incubated with 88 patient serum samples (*n* = 88). (A) Line graph showing raw signal intensities for RPS4Y1 AzK immobilized on DBCO-beads (orange) and COOH-beads (dark blue) across patient samples. To assess background signal levels, control beads were prepared by adding elution buffer instead of antigen to DBCO-beads (pink) and COOH-beads (light green). (B) Background signal intensities were subtracted for each data set to reveal the specific RPS4Y1 AzK-AAB binding signals for immobilization on DBCO-beads (orange) and COOH-beads (dark blue).

To assess the strength and direction of the relationship between signal intensities obtained from the two immobilization methods, we computed the Spearman correlation coefficient (r). The analysis revealed a strong positive correlation (*r* = 0.95, *p* < 0.0001), indicating that while signal intensities differ between methods, they scale proportionally. This relationship is visualized in a scatter plot (Figure 10A). It shows that as signal intensity increased in the random immobilization method, it also increased in the oriented immobilization method, reinforcing the strong positive correlation identified by the Spearman analysis. To further quantify this relationship, we performed linear regression analysis and added a trend line (Figure 10A). The resulting equation, *Y* = 1.684*X* + 3384, further supports the proportional relationship between the two methods. The slope (1.684) indicates that, on average, oriented immobilization yields 1.684 times higher binding signals than random immobilization. The intercept (3384 MFI) represents the predicted signal intensity for oriented immobilization when the signal from random immobilization is zero. The coefficient of determination (*R* ^2^ = 0.72) suggests that 72% of the variation in oriented immobilization signals can be explained by the signals from random immobilization, while the remaining 28% likely results from factors not accounted for by the linear model, such as sample biological complexity. To compare the overall distribution of signal intensities between the two immobilization methods, we generated a box-and-whisker plot using Tukey’s method (Figure 10B). The median signal intensity for oriented immobilization of RPS4Y1 AzK on DBCO-beads (8508 MFI) was 2.5-fold higher than that of random immobilization on COOH-beads (3471 MFI), confirming the enhanced binding sensitivity achieved with oriented immobilization. The plot also revealed greater variability and a broader distribution in oriented immobilization, as evidenced by a wider interquartile range (IQR) and longer whiskers, respectively compared to random immobilization. This suggests that while oriented immobilization enhances overall sensitivity, the extent of improvement varies between patient samples. Additionally, outliers in the oriented immobilization group exhibited notably higher signal intensities than those in the random immobilization group, indicating that some patient samples generated exceptionally strong responses when epitopes were optimally exposed. A Wilcoxon signed-rank test (a nonparametric test for paired data) confirmed that the difference in signal intensity between the two immobilization methods was statistically significant (*p* < 0.0001). Overall, these findings demonstrated that oriented immobilization of RPS4Y1 AzK significantly enhanced binding sensitivity compared to random immobilization, confirming that epitope accessibility was greater in the oriented setup, leading to more efficient AAB binding and enhanced sensitivity.

**Figure 10:**
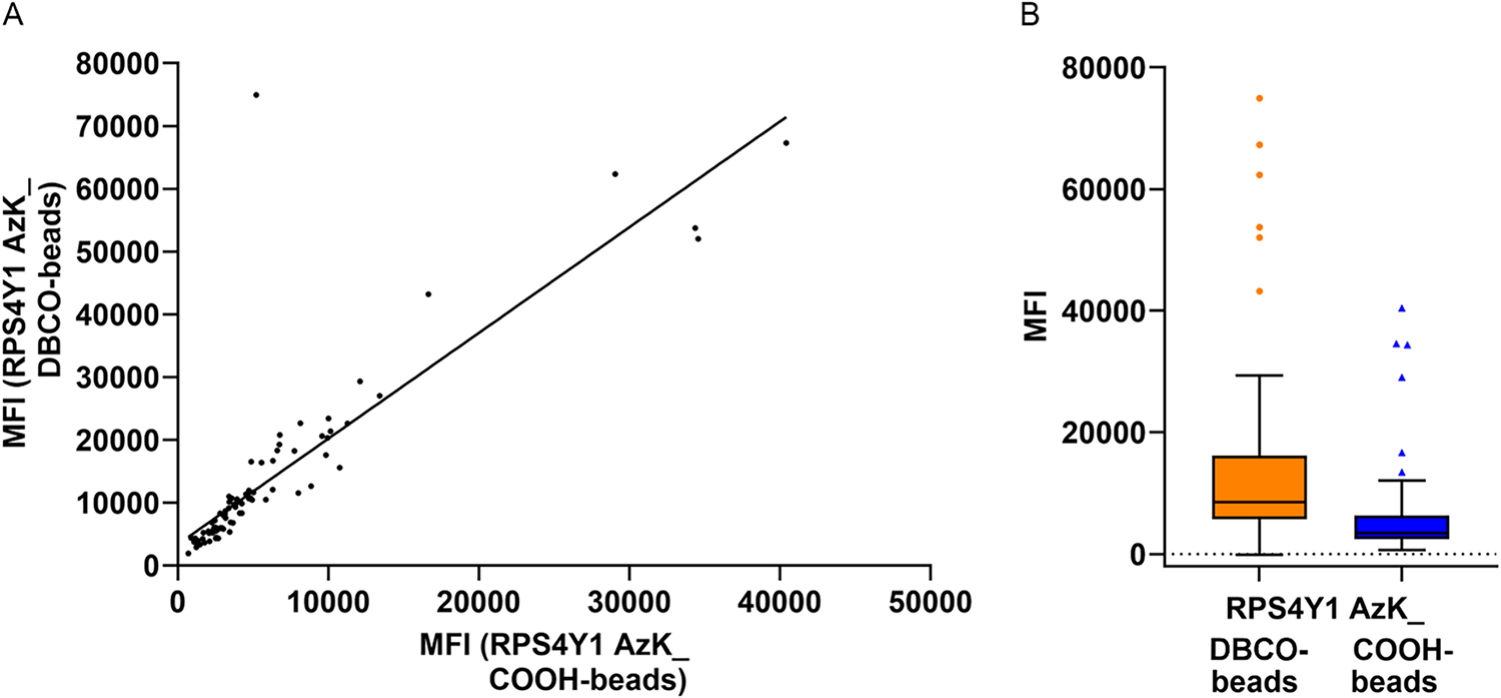
Statistical analysis confirms enhanced binding sensitivity achieved via oriented RPS4Y1 AzK immobilization. (A) Scatter plot illustrating the strong positive correlation (*r* = 0.95, *p* < 0.0001) between signal intensities from oriented immobilization of RPS4Y1 AzK on DBCO-beads (*Y*-axis) and its random immobilization on COOH-beads (*X*-axis) across patient samples. Each point represents an individual patient sample. Linear regression analysis (*Y* = 1.684*X* + 3384, *R* ^2^ = 0.72) indicates that, on average, oriented immobilization yields 1.684-fold higher signal intensities than random immobilization. (B) Box-and-whisker plot comparing signal distributions between oriented immobilization of RPS4Y1 AzK on DBCO-beads (orange) and its random immobilization on COOH-beads (dark blue). The boxes represent the interquartile range (IQR, 25th−75th percentile), the horizontal line within each box indicates the median, and whiskers extend up to 1.5 × IQR following Tukey’s method. Data points beyond this range are considered outliers and plotted as individual points. The median signal intensity for oriented immobilization was 2.5-fold higher than for random immobilization (8508 vs 3471 MFI). A Wilcoxon signed-rank test (*p* < 0.0001) confirmed that oriented immobilization significantly enhances binding sensitivity compared to random immobilization.

## 3 Conclusions

This study demonstrates that oriented antigen immobilization via genetic code expansion and click chemistry significantly enhances binding sensitivity on the Luminex platform compared to conventional amine coupling. Specifically, the oriented immobilization of RPS4Y1 AzK on DBCO-beads resulted in a 2.5-fold increase in binding sensitivity over random immobilization on COOH-beads. This finding confirms that SPAAC-mediated, site-specific conjugation of AzK-functionalized proteins ensures consistent antigen orientation and optimizes epitope accessibility, leading to more efficient AAB binding. In contrast, random immobilization via amine coupling can result in heterogeneous antigen orientations, potentially introducing steric hindrance and reducing AAB binding efficiency. However, our attempts to apply this strategy to HDAC3 AzK and RPS17 AzK antigens were impeded by their persistent nonspecific binding, which could not be mitigated through blocking strategies as was successful for RPS4Y1 AzK. This suggests that protein-specific properties, such as hydrophobicity, aggregation tendency, and stability during the solubilization process, may influence the effectiveness of blocking and, consequently, the success of site-specific immobilization. Future studies should investigate these factors to expand the applicability of this approach to a broader range of antigens, especially those produced as *E. coli* inclusion bodies. This could involve characterizing the surface properties of proteins in their solubilized states, optimizing solubilization and refolding conditions, or exploring alternative blocking strategies tailored to each antigen’s specific characteristics. Alternatively, expression in eukaryotic systems, such as mammalian cells, may be more suitable for antigens that are difficult to express in soluble form in *E. coli*. Despite these challenges, our findings underscore the potential of genetic code expansion and click chemistry in improving antigen presentation and AAB detection sensitivity on the Luminex platform. This approach may be extended to other bead-based or surface-immobilization platforms in biosensing and diagnostics, where high sensitivity and accuracy are essential.

## 4 Materials and Methods

### 4.1 Construction of Expression Plasmids

The expression plasmids pT7x31 ^52^ encoding human antigenic proteins were constructed in the following way. To generate inserts for the wild-type (wt) genes, the amino acid sequences of RPS17 (UniProt ID: P08708), RPS4Y1 (UniProt ID: P22090), and HDAC3 (UniProt ID: O15379) were modified by adding an N-terminal MRGS linker, a hexahistidine-tag (6H-tag) and a linker-adapter sequence (GSYLGDTIESSTHAS), as detailed in Büssow’s thesis.^51^ For amber mutant constructs, an analogous procedure was followed, with the additional insertion of an amber (am) stop codon at position 5, directly upstream of the 6H-tag. These modified sequences were back translated into DNA sequences, codon optimized for expression in *E. coli* and synthesized as clonal gene fragments by Twist Bioscience (South San Francisco, CA) (Table S1).

Chemically competent *E. coli* Top10F’ cells (Thermo Fisher Scientific Inc., Waltham, MA) were heat-shock transformed with clonal genes for *HDAC3, HDAC3am, RPS4Y, RPS4Yam, RPS17, and RPS17am*, regenerated and plated on Luria−Bertani (LB) agar plates containing 100 μg/mL ampicillin, as described elsewhere.^72^ Plasmids from selected ampicillin-resistant clones were isolated using the Wizard Plus SV Minipreps DNA Purification System (Promega Corporation, Madison, WI). The purified plasmids were digested with the restriction enzymes BglII/NdeI (Thermo Fisher Scientific Inc.) and the resulting DNA fragments were resolved on a 0.8% (w/v) agarose gel. Desired DNA fragments were excised from the gel and purified using the Wizard SV Gel and PCR Clean-Up kit (Promega Corporation).

Each purified digested insert was separately ligated into the BglII/NdeI-linearized pT7x31 expression vector using T4 DNA ligase (1 U) (Thermo Fisher Scientific Inc.). Chemically competent *E. coli* TOP10F’ cells were transformed with the ligation mixtures, regenerated, and plated on LB agar plates containing 50 μg/mL kanamycin. The resulting plasmids, pT7x31_*RPS17*, pT7x31_*RPS17am*, pT7x31_*RPS4Y1*, pT7x31_*RPS4Y1am*, pT7x31_*HDAC3*, and pT7x31_*HDAC3am*, were isolated and the genes of interest were sequence verified by Microsynth AG (Balgach, Switzerland). Subsequently, the chemically competent *E. coli* BL21­(DE3) expression strain (Merck KGaA, Darmstadt, Germany) was transformed with the sequence-verified plasmids and stored in 30% (v/v) glycerol at −80 °C.

### 4.2 Protein Production and Solubility Test

A 10 mL volume of LB medium containing 50 μg/mL kanamycin (LBKan) was inoculated with glycerol stocks of *E. coli* BL21­(DE3) carrying plasmids pT7x31_*RPS17*, pT7x31_*RPS17am*, pT7x31_*RPS4Y1*, pT7x31_*RPS4Y1am*, pT7x31_*HDAC3*, and pT7x31_*HDAC3am* and incubated overnight at 37 °C with vigorous shaking. The overnight cultures (ONC) were then added to 50 mL of fresh LBKan medium at a starting attenuance (D_600_) of 0.1. The cultures were incubated at 37 °C with shaking at 130 rpm until they reached a D_600_ of 0.8. At this point, 1 mL of each culture was collected as the before induction sample. Gene expression was induced with 1 mM IPTG, and the cultures were incubated at 28 °C for 18 h with shaking at 130 rpm. To produce the azide-labeled variant proteins, the ncAA H-l-Lys­(EO-N3)−OH*HCl (AzK; cat. no. HAA2080; Iris Biotech, Marktredwitz, Germany) was freshly prepared in sterile doubly distilled H_2_O (ddH_2_O) and added to the cultures at a final concentration of 5 mM at the time of IPTG induction.

To assess protein expression, a volume of the cell culture equivalent to a D_600_ of 0.8 was collected 18 h after induction with IPTG for wt proteins or with IPTG and AzK addition for AzK-containing proteins. After harvesting the cells by centrifugation at 17,000*g* for 5 min, the medium supernatant was discarded, and the cell pellets were resuspended in 80 μL of SDS sample buffer (50 mM Tris, 4% (v/v) glycerol, 1% (w/v) SDS, 100 mM dithiothreitol, 0.04% (w/v) bromophenol blue, 0.5% (v/v) β-mercaptoethanol). The samples were heated at 95 °C for 10 min. Insoluble debris was sedimented by centrifugation at 17,000*g* for 2 min, and the supernatant was analyzed by SDS-PAGE.

To evaluate the solubility of AzK-functionalized proteins, the remaining culture volume was divided into two equal portions and harvested by centrifugation at 8000 rpm (JA-10 rotor, Beckman Coulter Life Sciences, Indianapolis, IN) for 10 min. After harvesting, one portion was resuspended in 5 mL of lysis buffer (100 mM sodium phosphate (NaPi), 500 mM NaCl, 20 mM imidazole, 20 mM β-mercaptoethanol and 1% (v/v) Triton X-100), supplemented with 6 M urea, pH 8.0. Following a 2 h incubation at RT, insoluble debris was sedimented by centrifugation at 21000 rpm (JA-25.50 rotor, Beckman Coulter Life Sciences) for 30 min. An aliquot of the supernatant, representing the total protein fraction, was prepared for SDS-PAGE analysis.

The second portion was resuspended in 5 mL of lysis buffer, pH 8.0 and lysed by sonication (Branson Sonifier 250 Emerson Electric, St. Louis, MO) using 3 × 1 min pulses with 1 min cooling on ice between pulses. The lysate was centrifuged at 21,000 rpm for 30 min at 4 °C. An aliquot of the sonication supernatant, representing the soluble protein fraction, was mixed with SDS sample buffer and kept for SDS-PAGE analysis. For the insoluble fraction, the sonication pellet was resuspended in lysis buffer containing 6 M urea, pH 8, using a volume equal to that of the sonication supernatant to maintain consistent protein concentrations between the fractions. Following a 2 h incubation at RT, insoluble debris was removed by centrifugation at 21,000 rpm for 30 min. An aliquot of the supernatant, representing the solubilized insoluble protein fraction, was prepared for SDS-PAGE analysis.

### 4.3 Protein Purification by Immobilized Metal Affinity Chromatography (IMAC)

The solubilized insoluble protein fractions of RPS17 and RPS4Y1 were loaded onto Ni-NTA beads (Cube Biotech GmbH, Monheim, Germany), pre-equilibrated with lysis buffer containing 6 M urea, pH 8.0. The flow-through was collected by gravity and nonspecifically bound proteins were removed using wash buffer (lysis buffer with 6 M urea, pH 6.3). Target proteins were eluted with an elution buffer consisting of 100 mM NaPi, 500 mM NaCl and 6 M urea at pH 4.6. Since the elution buffer’s pH (4.6) is close to the pI of HDAC3 (5.2), its purification was based on imidazole concentration gradients instead of pH adjustment. Washing was performed with lysis buffer containing 6 M urea and 30 mM imidazole, pH 8, while elution was carried out with 100 mM NaPi, 500 mM NaCl, 6 M urea and 300 mM imidazole, pH 8.0. Elution fractions were mixed with SDS sample buffer for SDS-PAGE analysis. Fractions exhibiting the highest concentration and purity of target proteins, as determined by SDS-PAGE, were pooled for further analysis. To determine the protein concentrations of wt and AzK variants, a standard curve was generated using six different concentrations of bovine serum albumin (BSA). These BSA standards, along with purified protein samples, were loaded onto an SDS-PA gel. Following electrophoresis, the gel was imaged using the ChemiDoc Imaging System (Bio-Rad, Hercules, CA). Densitometric analysis was performed using Image Lab software (Bio-Rad), which quantified band intensities. A standard curve was constructed using known BSA concentrations and their corresponding band intensities, allowing the software to extrapolate the concentrations of target proteins. For storage, aliquots of purified proteins were kept at 4 °C until further use.

### 4.4 Conjugation Reaction with Fluorophore

IMAC purified wt and AzK variants of human antigenic proteins were individually conjugated with a 10-fold molar excess of the fluorophore DBCO-Cy3 (cat. no. CLK-A140; Jena Bioscience GmbH, Jena, Germany). The reaction mixtures were incubated at room temperature (RT) with shaking at 600 rpm for 2 h in the dark to facilitate SPAAC. The reaction was terminated by adding SDS sample buffer, followed by heating at 95 °C for 10 min.

### 4.5 Sodium Dodecyl Sulfate Polyacrylamide Gel Electrophoresis

Protein samples were separated on 4−12% polyacrylamide (PA) gels (NuPAGE Bis-Tris Mini Protein Precast Gels, Invitrogen, Waltham, MA). Electrophoresis was performed at 200 V for 40 min using NuPAGE MES-SDS running buffer (Thermo Fisher Scientific Inc.). Following electrophoresis, the SDS-PA gels were stained with InstantBlue Coomassie Protein Stain (Abcam plc., Cambridge, UK).

For fluorophore-conjugated proteins, gels were washed three times for 15 min each to remove unbound fluorophore. The fluorescent signal was visualized under UV illumination at 302 nm before staining with InstantBlue Coomassie Protein stain to confirm protein presence and integrity.

### 4.6 LC-ESI-MS/MS Analysis of Peptides Originating from Protease Digestion

Protein samples (HDAC3 wt, HDAC3 AzK, RPS4Y wt, RPS4Y AzK, RPS17 wt, and RPS17 AzK) were digested in-gel, with proteins first S-alkylated using iodoacetamide and then digested with chymotrypsin or trypsin (Promega Corporation). The resulting peptides were loaded onto a nanoEase C18 column (nanoEase M/Z HSS T3 Column, 100 Å, 1.8 μm, 300 μm x 150 mm; Waters Corporation, Milford, MA) with 0.1% (v/v) formic acid in water as the aqueous solvent. Peptides were separated using a 30 min gradient elution (solvent A: 0.1% (v/v) formic acid in water; solvent B: 0.1% (v/v) formic acid in 80% (v/v) acetonitrile in water) at a flow rate of 6 μL/min. Detection was performed with an Orbitrap Exploris 480 mass spectrometer (Thermo Fisher Scientific Inc.) equipped with a standard H-ESI source, operating in positive ion mode. Data-dependent acquisition (DDA) was used, automatically switching to MS/MS mode for eluting peaks. MS scans were recorded over a mass range of 350−1200 Da, and the 20 most intense peaks were selected for fragmentation. Instrument calibration was performed using the Pierce FlexMix Calibration Solution (Thermo Fisher Scientific Inc.). Raw data files were analyzed using PEAKS software (Bioinformatics Solutions Inc., Waterloo, Canada), performing MS/MS ion searches against target proteins and an *E. coli* BL21­(DE3) database.

### 4.7 Protein Immobilization on DBCO-Agarose Beads

Twenty μL of DBCO-agarose beads (50% aqueous suspension; cat. no. CLK-1034−2; Jena Bioscience, Jena, Germany) were washed twice with 200 μL of ddH_2_O, followed by equilibration with 100 mM NaPi buffer, pH 8.0 through four washes (200 μL each). 40 μL of RPS17 AzK and RPS4Y1 AzK were incubated with DBCO-agarose beads in elution buffer at final concentrations of 1.6 and 1.9 mg/mL, respectively, for 24 h at 22 °C with shaking at 1000 rpm to allow SPAAC conjugation. The reaction conditions resulted in a 63-fold molar excess of DBCO groups relative to RPS4Y1 AzK and a 43-fold molar excess relative to RPS17 AzK. To assess nonspecific binding, wt RPS17 and RPS4Y1 were incubated with DBCO-agarose beads under identical conditions as negative controls. Additionally, 40 μL of wt and AzK protein variants were incubated without DBCO-agarose beads to evaluate protein stability. After incubation, the reaction mixtures were centrifuged at 17,000*g* for 5 min, and supernatants (40 μL) were collected and prepared for SDS-PAGE analysis. Beads were washed 5x with 40 μL of 100 mM NaPi, pH 8.0 containing 500 mM NaCl, 20 mM β-mercaptoethanol, 1% (v/v) Triton X-100, and 20 mM imidazole. Collected wash fractions (40 μL) were also prepared for SDS-PAGE analysis. To calculate immobilization efficiency, protein concentrations of the input material and the postincubation supernatant and wash fractions were determined spectrophotometrically (NanoDrop 2000 spectrophotometer; Thermo Fisher Scientific Inc.).

#### 4.7.1 Immunodetection of Immobilized Protein on DBCO-Agarose Beads

To validate protein immobilization, 500 μL of blocking solution (SuperBlock Blocking Buffer; Thermo Fisher Scientific Inc.) were added to DBCO-agarose beads immobilized with RPS4Y1 AzK and incubated for 1 h at RT. Beads were then washed 3x with 500 μL of PBST (PBS containing 0.1% (v/v) Tween 20). Next, the beads were incubated with 200 μL of rabbit anti-6-His antibody (cat. no. SAB4301134; Merck KGaA), diluted 1:1000 in blocking solution, for 1 h at RT. After three additional washes with 500 μL of PBST, the beads were incubated with 200 μL of antirabbit IgG−peroxidase antibody produced in goat (cat. no. A0545; Merck KGaA), diluted 1:30,000 in blocking solution, for 1 h at RT. The same protocol was applied to DBCO-agarose beads incubated with RPS4Y1 wt, which served as a negative control. Beads were washed three times with 500 μL of PBST, and the enzymatic reaction was initiated by adding 3,3′,5,5′-tetramethylbenzidine (TMB) substrate solution to each bead type. The reaction was stopped by adding 1 M sulfuric acid, and absorbance at 450 nm was measured using a microplate reader (BioTek Synergy H1, Agilent, Santa Clara, CA).

### 4.8 Functionalization of Luminex beads

#### 4.8.1 Functionalization of Luminex Beads with DBCO Groups

To functionalize carboxylated polystyrene beads (MagPlex-C Microspheres, 1 mL; cat. no. MC10XXX-01; Luminex Corporation, Austin, TX) with DBCO groups, 8.5 μL of bead stock (containing ∼10^5^ beads) per bead type was pipetted into individual wells of a 96-deep-well plate (Deepwell Protein LoBind Plates, 500 μL; Eppendorf, Hamburg, Germany). First, 100 μL of 100 mM NaH_2_PO_4_, pH 6.2 was added to the beads, followed by vortex mixing (Minishaker MS2; IKA-Werke GmbH & Co. KG, Staufen im Breisgau, Germany) for 20 s and sonication in an ultrasonic cleaning bath (Transsonic T470/H; Elma Schmidbauer GmbH, Singen, Germany) for 20 s. The plate was centrifuged at 2000 rpm for 1 min using a Heraeus Megafuge 40R Refrigerated Benchtop Centrifuge (Thermo Fisher Scientific Inc., Waltham, MA). After centrifugation, the plate was placed on a magnetic plate holder (Luminex Corporation) to allow the beads to settle at the bottom of the wells. The supernatant was carefully aspirated using a pipet. Next, 80 μL of 100 mM NaH_2_PO_4_, pH 6.2 was added to the beads, and the vortex mixing and sonication steps were repeated for 20 s each. Fresh solutions of 1-ethyl-3-(3-(dimethylamino)­propyl)­carbodiimide hydrochloride (EDC; Pierce EDC, No-Weigh Format; cat. no. 22980; Thermo Fisher Scientific Inc.) and sulfo-*N*-hydroxysulfosuccinimide (sulfo-NHS, No-Weigh Format; cat. no. 24510; Thermo Fisher Scientific Inc.) were prepared at 50 mg/mL in ddH_2_O immediately prior to use. To activate carboxyl (COOH) groups on the beads, 10 μL of 50 mg/mL EDC was added to each well, and the beads were vortexed for 20 s. EDC reacts with COOH groups to form a reactive intermediate, which can quickly react with primary amines to form stable amide bonds. However, this intermediate is unstable in aqueous solutions. To stabilize the reactive intermediate and increase reaction efficiency, 10 μL of 50 mg/mL sulfo-NHS was added before EDC addition, followed by vortex mixing the beads for 20 s. Sulfo-NHS reacts with EDC-activated COOH groups to form amine-reactive sulfo-NHS esters, which are more stable and enhance coupling efficiency during subsequent reactions with primary amines. The plate was incubated at RT in the dark for 20 min on a plate shaker (Titramax 1000; Heidolph Scientific Products GmbH, Schwabach, Germany) at 700 rpm, with an additional vortex mixing step after 10 min.

Meanwhile, a stock solution of sulfo DBCO-PEG4-amine linker (cat. no. BP-23310; BroadPharm, San Diego, CA) was prepared at a concentration of 50 mg/mL in ddH_2_O. From this stock solution, a series of six dilutions were prepared using 50 mM 2-(N-morpholino)­ethanesulfonic acid (MES buffer, pH 5.0; Merck KGaA). The first dilution was prepared by mixing 4 μL of stock solution with 396 μL of MES buffer, resulting in a linker concentration of 500 μg/mL. The second, third and fourth dilutions were prepared by sequentially halving the concentration, resulting in concentrations of 250 μg/mL, 125 μg/mL and 62.5 μg/mL, respectively. The last two dilutions were made by further diluting the preceding concentration by a factor of 4, resulting in final concentrations of 15.6, and 3.9 μg/mL.

After activation of COOH groups, to remove excess EDC and sulfo-NHS reagents, the plate was centrifuged at 2000 rpm for 1 min, placed on the magnetic separator, and the supernatant was aspirated using a pipet. 200 μL of MES buffer, pH 5.0 was added to the beads, followed by vortex mixing for 20 s and sonication for 20 s. The plate was centrifuged again at 2000 rpm for 1 min, placed on the magnetic separator and the supernatant was aspirated. This washing step with MES buffer was repeated twice for a total of three washes. Following the washes, 200 μL of each sulfo DBCO-PEG4-amine linker dilution (500, 250, 125, 62.5, 15.6, and 3.9 μg/mL) was added to separate wells containing the activated beads (each well containing a distinct bead type). Beads were vortexed for 20 s to ensure thorough mixing, and the plate was incubated in the dark at RT for 2 h on a plate shaker at 600 rpm to facilitate amine coupling. After incubation, to remove unbound linker, the plate was centrifuged at 2000 rpm for 1 min, placed on the magnetic separator, the supernatant was aspirated, and the beads were washed twice with 200 μL of MES buffer, pH 5.0. Each wash involved vortex mixing for 20 s, sonication for 20 s, centrifugation at 2000 rpm for 1 min, magnetic separation, and aspiration of the supernatant. To quench any remaining reactive intermediates (amine-reactive sulfo-NHS esters), 200 μL of 1 M ethanolamine, pH 8.5 was added to the beads. The plate was vortexed briefly, and passivation was carried out by incubating the beads at RT in the dark for 2 h on a plate shaker at 600 rpm. After incubation, excess ethanolamine was removed by plate centrifugation at 2000 rpm for 1 min followed by magnetic separation and aspiration of the supernatant. The beads were washed three times with 200 μL of phosphate-buffered saline (PBS, pH 7.4; Thermo Fisher Scientific Inc.) containing 0.05% (v/v) Tween 20 (Merck KGaA) (PBS-T). Each wash involved vortex mixing, sonication, centrifugation at 2000 rpm for 1 min, magnetic separation, and supernatant aspiration. After the final wash, 200 μL of PBS, pH 7.4 was added to each well to resuspend the beads. The plate was sealed and stored at 4 °C in the dark to preserve the functionalized beads.

#### 4.8.2 Functionalization of Luminex Beads with COOH Groups

To ensure consistency in spacer chemistry for comparison with DBCO-functionalized beads, carboxylated Luminex MagPlex beads were functionalized with an amino-PEG4-COOH linker (amino-PEG4-acid, cat. no. BP-20423; BroadPharm) following a procedure analogous to that used for DBCO-functionalized beads.

The COOH groups on the beads were activated using EDC and sulfo-NHS, as described previously, and the activated beads were washed three times with MES buffer, pH 5.0. A stock solution of the amino-PEG4-COOH linker was prepared at a concentration of 20 mg/mL. From this stock solution, a series of dilutions were made using MES buffer, pH 5.0. In the first functionalization experiment, the initial dilution was prepared by mixing 2 μL of the stock solution with 398 μL of MES buffer, resulting in a linker concentration of 100 μg/mL. Two additional dilutions were then prepared by sequentially halving the concentration, yielding final concentrations of 50 μg/mL and 25 μg/mL. For the second functionalization experiment, to evaluate the effect of increased linker availability on functionalization efficiency, higher linker concentrations of 250 μg/mL and 500 μg/mL were tested.

The amine coupling reaction between the activated COOH groups on the beads and the amino-PEG4-COOH linker was carried out by incubating the beads with the linker solutions in the dark at RT for 2 h on a plate shaker set to 600 rpm. Following incubation, unbound linker was removed by washing the beads two times with MES buffer, pH 5.0. Each wash included vortex mixing, sonication, centrifugation, and magnetic separation, as previously described. To quench any remaining reactive intermediates, a passivation step was performed by adding 1 M ethanolamine, pH 8.5 to the beads and incubating them in the dark at RT for 2 h on a plate shaker at 600 rpm. Excess ethanolamine was removed through three washes with PBS-T, with each wash involving vortex mixing, sonication, centrifugation, magnetic separation and aspiration of the supernatant. Finally, the COOH-functionalized beads were resuspended in 200 μL of PBS, pH 7.4 and stored at 4 °C in the dark to preserve their functionality.

#### 4.8.3 Confirmation of Functionalization of Luminex Beads

The successful modification of carboxylated MagPlex beads with DBCO groups was verified using an azide-PEG3-biotin conjugate (cat. no. 762024; Merck KGaA). Prior to adding the conjugate for verification, the beads were prepared as follows. According to the Luminex Cookbook,^9^ approximately 1000 beads per bead type are required per reaction (well), with an additional 10% margin for pipetting variability. The assay was designed to include a total of 10 reactions (five different conjugate concentrations tested in duplicates), requiring at least 11,000 beads per bead type (10 wells × 1.1 × 1000 beads). Beads were stored at a concentration of 10^5^ beads per bead type in 200 μL of PBS, pH 7.4. To obtain the required bead count, 22 μL of beads per type was taken, corresponding to approximately 11000 beads. The beads were pooled by transferring 22 μL of each bead type into a single safe-lock tube (Eppendorf). The pooled beads were centrifuged at 3500 rpm for 3 min, and the supernatant was carefully removed using a pipet while holding the tube against a magnetic rack for tubes (DynaMag-2 magnet; Thermo Fisher Scientific Inc.) to minimize bead loss. The resulting bead pellet was resuspended in 330 μL of MES buffer, pH 5.0, to achieve the appropriate working concentration for the assay. This resuspension volume was calculated based on the 10 planned reactions, each requiring 30 μL of bead suspension per well, with a 10% pipetting margin (10 wells × 1.1 × 30 μL = 330 μL). The bead suspension was then aliquoted into a round-bottom clear, polystyrene 96-well plate (Corning 96 Well Clear Polystyrene Microplate; Merck KGaA), with 30 μL of bead suspension dispensed into each of the 10 wells.

In the meantime, a stock solution of azide-PEG3-biotin at a concentration of 50 mg/mL was used to prepare five linker dilutions in MES buffer, pH 5. The first dilution was prepared by mixing 2 μL of stock solution with 198 μL of MES buffer, resulting in a final linker concentration of 500 μg/mL. Subsequent dilutions were prepared by sequentially halving the concentration, resulting in solutions of 250 μg/mL, 125 μg/mL, and 62.5 μg/mL. A control (blank) consisting of MES buffer without linker (0 μg/mL) was also included.

The 96-well plate containing the beads was placed on a magnetic separator to allow the beads to assemble at the bottom of the wells. The supernatant was removed by inverting the plate forcefully over a sink and tapping it onto tissue paper. The plate was removed from the magnet separator, and 50 μL of each azide-PEG3-biotin conjugate dilution was added to the wells containing DBCO-functionalized beads. For the control well, 50 μL of MES buffer was added instead of linker solution. The reaction was gently mixed by pipetting up and down, avoiding foam formation. The plate was sealed with a plate sealer (Corning microplate sealing tape; Merck KGaA) and incubated in the dark at RT for 2 h on a plate shaker set to 700 rpm to allow the SPAAC reaction to proceed. After incubation, the plate was placed on a magnetic separator, and the supernatant was removed by inverting the plate over a sink. The plate was removed from the separator, and 200 μL of PBS-T was added to each well. This washing process was repeated three times, with no wash buffer added after the final step.

To confirm biotin incorporation, a solution of Streptavidin-R-Phycoerythrin Conjugate (SA-PE; cat. no. S21388; Thermo Fisher Scientific Inc.) was prepared at 10 μg/mL from a 1000 μg/mL stock in PBS containing 1% (w/v) bovine serum albumin (BSA; Merck KGaA), 0.05% (w/v) sodium azide (NaN_3_, Avantor, Inc., Radnor, PA), pH 7.4 (assay buffer). 50 μL of SA-PE solution was added to each well and the reaction was mixed by gently pipetting up and down, avoiding foam formation. The plate was sealed with a plate sealer and incubated for 30 min at RT in the dark on a plate shaker set to 700 rpm. After incubation, the plate was placed on a magnetic separator and the supernatant was removed as above. The beads were washed three times with 100 μL of PBS-T, ensuring no buffer was added after the final wash. After the final washing step, the plate was removed from the magnetic separator and 100 μL of assay buffer was added to each well to resuspend the beads. The suspension was mixed gently by pipetting up and down, avoiding foam formation. The beads were analyzed using a FLEXMAP 3D Instrument System (Luminex Corporation).

To confirm the successful functionalization of COOH-functionalized beads, an amine-PEG3-biotin conjugate (EZ-Link Amine-PEG3-Biotin; cat. no. 21347; ThermoFisher Scientific Inc.) was used. Before adding the biotin-conjugate for verification, the COOH groups derived from the coupled amino-PEG4-COOH linker were activated in the following way. The storage buffer was exchanged by centrifuging the storage plate, placing it on a magnetic holder, and aspirating the supernatant. The beads were then washed twice with 100 μL of 100 mM NaH_2_PO_4_, pH 6.2. After washing, 80 μL of NaH_2_PO_4_, pH 6.2 was added to the beads. To activate the COOH groups for amine coupling with the amine-PEG3-biotin linker, 10 μL each of freshly prepared sulfo-NHS and EDC were added to the wells containing the beads. The plate was incubated at RT in the dark for 20 min at 700 rpm. Following activation, the beads were washed three times with 200 μL of MES buffer, pH 5.0 to remove residual activation reagents and prepare the beads for the coupling reaction.

The confirmation assay for COOH-functionalized beads included a total of 10 reactions (five different conjugate concentrations tested in duplicates), requiring at least 11,000 beads per bead type. The beads were prepared following the same procedure as described for the confirmation assay with DBCO-functionalized beads. Briefly, to obtain the required bead count, 22 μL of beads per bead type was taken. The beads were pooled by transferring 22 μL of each bead type into a single Eppendorf tube. The pooled beads were centrifuged, and the supernatant was carefully removed. The resulting bead pellet was resuspended in 330 μL of MES buffer, pH 5.0, to achieve the appropriate working concentration for the assay. The bead suspension was then aliquoted as 30 μL per well across 10 wells.

A stock solution of amine-PEG3-biotin (50 mg/mL) was diluted in MES buffer, pH 5 to prepare five serial dilutions. The first dilution (500 μg/mL) was made by mixing 2 μL of stock solution with 198 μL of MES buffer. Subsequent dilutions were prepared by sequentially halving the concentration, yielding solutions with final concentrations of 250, 125, and 62.5 μg/mL. A blank control (0 μg/mL) containing only MES buffer was also included.

A 96-well plate containing 30 μL bead aliquots was placed on a magnetic separator, the supernatant was removed, and 50 μL of each amine-PEG3-biotin dilution was added to the wells containing COOH-functionalized beads. For the control well, 50 μL of MES buffer was added instead of the linker solution. The plate was gently mixed and incubated at RT in the dark for 2 h on a plate shaker (700 rpm) to allow amine coupling. After incubation, the beads were washed three times with 200 μL of PBS-T, with no wash buffer added after the final step. The remainder of the protocol, including incubation with SA-PE, washing with PBS-T, addition of assay buffer, and analysis using the FLEXMAP 3D Instrument System followed the same procedure as described for confirming the functionalization of DBCO-functionalized beads.

### 4.9 Protein Immobilization

#### 4.9.1 Protein Immobilization on DBCO-Functionalized Beads

For each type of carboxylated MagPlex beads, 8.5 μL of bead stock (approximately 10^5^ beads) was aliquoted into separate wells of a 96-deepwell Eppendorf plate. Beads were functionalized with 250 μg/mL of sulfo DBCO-PEG4-amine, following the procedure described in the “Functionalization of Luminex beads with DBCO groups” section.

Based on the Luminex Cookbook, 5 μg of protein is required to couple 10^6^ beads, which translates to 0.5 μg of protein for coupling 10^5^ beads. To prepare the protein dilutions for coupling, the required amounts of protein and MES buffer, pH 5 were calculated to achieve a final concentration of 0.5 μg of protein per 10^5^ beads in a total reaction volume of 100 μL. The target proteins for coupling were AzK-functionalized antigens (HDAC3, RPS4Y1, and RPS17). To evaluate the specificity of coupling process, wt antigens HDAC3, RPS4Y1, and RPS17 were also used. A negative control consisting only of MES buffer (without protein) was included for additional validation.

The 96-deepwell plate containing 10^5^ DBCO-functionalized beads per well was centrifuged at 2000 rpm for 1 min (to remove PBS as a storage buffer). The plate was placed on a magnetic separator to allow the beads to assemble at the bottom of the wells, and the supernatant was removed by aspiration with a pipet. To each well, 100 μL of MES buffer, pH 5 was added. The beads were vortexed for 20 s and sonicated for 20 s. Next, 100 μL of the prepared protein solutions (or MES buffer for the negative control) was added to the respective well. Each protein variant was coupled to one bead type, with a total of seven bead types (six antigens and one MES buffer control). The beads were vortexed for 20 s to ensure thorough mixing, and the plate was incubated in the dark at RT for 2 h, mixing at 600 rpm on a plate shaker to facilitate the SPAAC reaction. Following incubation, the plate was centrifuged at 2000 rpm for 1 min, placed on a magnetic separator, and the supernatant was removed by aspiration. To each well, 200 μL of PBS, containing 0.1% (w/v) BSA, 0.02% (v/v) Tween 20 and 0.05% (w/v) NaN_3_, pH 7.4 (PBS-TBN) was added, followed by vortex mixing for 20 s and sonication for 20 s. The plate was centrifuged again at 2000 rpm for 1 min, placed on the magnetic separator and the supernatant was removed. These washing steps with PBS-TBN were repeated twice, for a total of three washes. Finally, 200 μL of assay buffer was added to each well. The coupled beads were stored at 4 °C in the dark.

#### 4.9.2 Protein Immobilization on COOH-Functionalized Beads

To generate COOH-functionalized beads, carboxylated MagPlex beads were functionalized with 250 μg/mL of amino-PEG4-COOH, following the procedure described in the “Functionalization of Luminex beads with COOH groups” section.

For the immobilization process, a 96-deepwell plate containing 10^5^ COOH-functionalized beads per well was centrifuged at 2000 rpm for 1 min. The plate was then placed on a magnetic separator to allow the beads to assemble at the bottom of the wells, and the supernatant (PBS as a storage buffer) was carefully aspirated. To prepare the COOH-functionalized beads for activation, 100 μL of 100 mM NaH_2_PO_4_, pH 6.2 was added to each well, followed by vortex mixing and sonication for 20 s each. The plate was centrifuged, placed on the magnetic separator, and the supernatant was aspirated. Next, 80 μL of NaH_2_PO_4_, pH 6.2 was added to the beads, and the vortex mixing and sonication steps were repeated. To activate COOH groups on the beads fresh solutions of EDC and sulfo-NHS were prepared at 50 mg/mL, following the procedure described earlier. Briefly, sulfo-NHS and EDC were sequentially added to each well, and the plate was incubated in the dark at RT for 20 min at 700 rpm. For experiments without EDC/sulfo-NHS activation, 100 μL of NaH_2_PO_4_, pH 6.2 was added instead and incubated under the same conditions.

To prepare protein solutions for coupling, the required amounts of proteins and MES buffer, pH 5 were calculated to achieve a final concentration of 0.5 μg of protein per 10^5^ beads in a total reaction volume of 100 μL. Wild-type antigens (HDAC3, RPS4Y1, and RPS17) were used as the target proteins for coupling. To evaluate the specificity of coupling process, the wt antigens were also tested without EDC/sulfo-NHS activation. A negative control consisting only of MES buffer (without protein) was included to further validate the assay.

Following activation of the COOH groups, the plate was centrifuged at 2000 rpm for 1 min, placed on the magnetic separator, and the supernatant was aspirated. Beads were washed three times with 200 μL of MES buffer, pH 5.0. Each wash involved vortex mixing for 20 s, sonication for 20 s, centrifugation at 2000 rpm for 1 min, magnetic separation, and supernatant aspiration. After the final wash, 100 μL of MES buffer was added to each well, followed by vortex mixing and sonication for 20 s each. Next, 100 μL of the prepared protein solutions (or MES buffer for the negative control) were added to the respective wells containing the COOH-functionalized beads (with or without EDC/sulfo-NHS activation). The beads were vortexed for 20 s, and the plate was incubated in the dark at RT for 2 h on a plate shaker set at 600 rpm to facilitate amine coupling. After incubation, the plate was centrifuged, placed on the magnetic separator, and the supernatant was aspirated. Beads were then washed three times with 200 μL of PBS-TBN. Each wash included vortex mixing, sonication, centrifugation, magnetic separation, and supernatant aspiration. After the final wash, 200 μL of assay buffer was added to each well. The coupled beads were stored at 4 °C in the dark until performing confirmation of the coupling process.

#### 4.9.3 Confirmation of Protein Immobilization on DBCO-Functionalized Beads

The successful immobilization of AzK-labeled antigens (HDAC3, RPS4Y1, and RPS17) on DBCO-functionalized beads was verified using a mouse anti histidine tag:biotin antibody, clone AD1.1.10 (cat. no. MCA1396B; Bio-Rad Laboratories, Inc., Hercules, CA). Prior to adding the anti-6H-tag-biotin antibody for verification, the beads were prepared as follows.

As already noted above approximately 1000 beads per bead type are required per reaction (well), with an additional 10% margin to account for pipetting deviations. For a total of 16 reactions (eight different antibody concentrations tested in duplicates), a minimum of 18,000 beads per bead type was needed (16 wells x 1.1 × 1000 beads). Beads immobilized with target proteins were stored at a concentration of 10^5^ beads per bead type in 200 μL of assay buffer. To obtain the required bead count, 36 μL of beads per type was taken, corresponding to approximately 18,000 beads. These beads were pooled by transferring 36 μL of each bead type into a single safe-lock tube. The pooled beads were centrifuged at 3500 rpm for 3 min, and the supernatant was carefully removed using a pipet while holding the tube on a magnetic rack to minimize bead loss. The bead pellet was resuspended in 528 μL of assay buffer, to achieve the working concentration required for the assay. This resuspension volume was calculated based on the planned 16 reactions, each requiring 30 μL of bead suspension per well, with a 10% pipetting margin (16 wells × 1.1 × 30 μL = 528 μL). The bead suspension was then aliquoted into a round-bottom, clear 96-well plate, with 30 μL of bead suspension dispensed into each of the 16 wells.

In parallel, a stock solution of anti-6H-tag-biotin antibody at 1 mg/mL was used to prepare eight serial dilutions in assay buffer. The first dilution was made by mixing 2.4 μL of the stock solution with 237.6 μL of assay buffer, resulting in a final concentration of 10 μg/mL. The second dilution was prepared by performing a 2.5-fold dilution of the first, yielding a concentration of 4 μg/mL. Subsequent dilutions were prepared by sequentially halving the concentration, resulting in solutions with final concentrations of 2, 1, 0.5, 0.25, and 0.13 μg/mL. A control (blank) consisting of assay buffer without antibody (0 μg/mL) was also included.

The 96-well plate containing the protein-immobilized beads was placed on a magnetic separator to allow the beads to assemble at the bottom of the wells. The supernatant was removed by inverting the plate forcefully over a sink and tapping it onto tissue paper. The plate was removed from the magnetic separator, and 50 μL of each anti-6H-tag-biotin antibody dilution was added to the respective wells. For the control well, 50 μL of assay buffer was added instead of the antibody solution. The reactions were gently mixed by pipetting up and down, ensuring no foam formation. The plate was sealed with a plate sealer and incubated in the dark at RT for 1 h on a plate shaker set to 700 rpm. Following incubation, the plate was placed on a magnetic separator, and the supernatant was removed by inverting the plate over a sink. Each well was then washed with 200 μL of PBS-T. The washing process was repeated three times, with no wash buffer added after the final wash.

To confirm biotin incorporation, a SA-PE solution was prepared at a concentration of 10 μg/mL by diluting a 1000 μg/mL stock solution in assay buffer. A volume of 50 μL of SA-PE solution was added to each well and the reactions were mixed gently by pipetting up and down, avoiding foam formation. The plate was sealed with a plate sealer and incubated for 30 min at RT in the dark on a plate shaker set to 700 rpm. After incubation, the plate was placed on a magnetic separator and the supernatant was removed as described above. The beads were washed three times with 100 μL of PBS-T, ensuring no wash buffer was added after the final wash. After the final washing step, the plate was removed from the magnetic separator and 100 μL of assay buffer was added to each well to resuspend the beads. The suspension was gently mixed by pipetting up and down, avoiding foam formation. The beads were then analyzed using a FLEXMAP 3D Instrument System.

#### 4.9.4 Confirmation of Protein Immobilization on COOH-Functionalized Beads

The procedure for confirming the immobilization of wt antigens (HDAC3, RPS4Y1, and RPS17) on COOH-functionalized beads followed the protocol described in the section “Confirmation of protein immobilization on DBCO-functionalized beads”. The only difference was the use of COOH-functionalized beads in place of DBCO-functionalized beads. All steps, including bead preparation, anti-6H-tag-biotin antibody preparation, SA-PE preparation, incubation, and washing, were performed in the same manner.

### 4.10 Blocking of Beads Prior Protein Immobilization

Blocking solutions were prepared using BSA and casein at various concentrations. Final BSA concentrations were 1%, 0.5%, and 0.1% (all w/v), with an additional 1% BSA solution supplemented with 0.05% (v/v) Tween 20. Casein (Merck KGaA) was prepared at final concentrations of 1% and 0.05% (w/v), along with a 0.5% casein solution containing 0.05% (v/v) Tween 20. All blocking solutions were prepared in PBS, pH 7.4. Following functionalization with reactive linkers, beads were collected by centrifugation at 2000 rpm for 1 min. The plate was then placed on a magnetic separator, and the supernatant was aspirated using a pipet. 200 μL of each blocking solution and PBS, pH 7.4 was added to the beads in the wells, followed by vortex mixing for 20 s. The plate was incubated in the dark at RT for 4 h on a plate shaker at 700 rpm. After incubation, to remove excess blocking agent, the plate was centrifuged at 2000 rpm for 1 min, placed on the magnetic separator and the supernatant was aspirated. The beads were then washed twice with 200 μL of PBS, pH 7.4. Each wash step included vortex mixing (20 s), sonication (20 s), centrifugation (2000 rpm,1 min), magnetic separation, and aspiration of the supernatant. Following the final wash, antigen solutions prepared in their elution buffers were added to the beads, and antigen coupling was performed as described previously. To assess antigen coupling efficiency after the blocking step, immobilization confirmation was conducted as outlined in “Confirmation of protein immobilization on DBCO-functionalized beads”. The only modification in this experiment was the use of a PE conjugated detection antibody, PE Anti-6X His tag antibody [AD1.1.10] (cat. no. ab72467; Abcam, Cambridge, UK). The anti-6H-tag-PE detection antibody was diluted from a 100 μg/mL stock solution to a final concentration of 10 μg/mL in assay buffer and tested in duplicate.

### 4.11 Assay with Patient Serum Samples

Purified IgG eluates from serum samples were prepared in a 96-well PCR plate (PCR plates, CFX style, clear, 0.1 mL, Avantor, Inc.) at a concentration of 0.2 mg/mL in Melon Gel Purification Buffer (Thermo Fisher Scientific Inc.). The purified IgG samples were diluted by mixing 30 μL of IgG samples with 30 μL of 2X assay buffer, resulting in a final IgG concentration of 0.1 mg/mL.

DBCO-and COOH-beads coupled with RPS4Y1 AzK antigen in elution buffer, along with control beads (DBCO- and COOH-beads incubated with elution buffer alone, without antigen), were prepared as described earlier. Briefly, after antigen coupling, the beads were stored at a concentration of 10^5^ beads per bead type in 200 μL of assay buffer. The assay consisted of 88 reactions (corresponding to 88 serum samples), requiring at least 968000 beads per bead type (88 wells × 1.1 × 1000 beads). To obtain the required bead count, 193.6 μL of each bead type was taken. The pooled beads were centrifuged at 3500 rpm for 3 min, and the supernatant was removed. The resulting bead pellet was then resuspended in 2904 μL of assay buffer to achieve the assay working concentration (88 wells × 1.1 × 30 μL = 2904 μL). The bead suspension was aliquoted into a 96-well plate, dispensing 30 μL into each of the 88 wells. The plate was placed onto a magnetic separator to allow beads to settle, and the supernatant was removed by forcefully inverting the plate over a sink. The plate was then removed from the magnet, and 50 μL of diluted IgG sample was added to each well. The reaction was mixed by pipetting up and down. The plate was sealed with a plate sealer foil and incubated for 2 h at RT in the dark, shaking at 700 rpm. Following incubation, the plate was placed on a magnetic separator, and the supernatant was removed. Each well was washed three times with 100 μL of PBS-T.

For detection, a 1:1 mixture of two R-Phycoerythrin (PE)-labeled antibodies was used: R-Phycoerythrin AffiniPure F­(ab’)_2_ Fragment Goat Anti-Human IgG, Fcγ fragment specific (cat. no. 109−116−098; Jackson ImmunoResearch Laboratories Inc., West Grove, PA) and R-Phycoerythrin AffiniPure F­(ab′)_2_ Fragment Goat Anti-Human IgG, F­(ab′)_2_ fragment specific (cat. no. 109−116−097; Jackson ImmunoResearch Laboratories Inc.). A 1:1 mixture of the two detection antibodies was prepared in assay buffer at a concentration of 2.5 μg/mL of each antibody (1:200), ending up in total with 5 μg/mL antibody. This means that per 1000 μL detection solution 5 μL of Fcγ fragment specific antibody (stock = 0.5 mg/mL) and 5 μL of F­(ab′)_2_ fragment specific antibody (stock = 0.5 mg/mL) were added to 990 μL of assay buffer. 50 μL of the antibody detection solution was added to each well and mixed by pipetting. The plate was sealed and incubated for 1 h at RT in the dark, shaking at 700 rpm. After incubation, the plate was placed on the magnetic separator, and the supernatant was removed by inverting the plate. Wells were then washed three times with 100 μL of PBS-T. Finally, 100 μL of assay buffer was added to each well, and the suspension was mixed by pipetting. The assay was analyzed using the FLEXMAP 3D system and the collected data were subsequently subjected to statistical analysis.

#### 4.11.1 Statistical Analysis

All statistical analyses were performed using GraphPad Prism (version 10.4.1, GraphPad Software, Boston, MA). Background signals were subtracted from each data set before analysis. To assess whether the data followed a Gaussian (normal) distribution, a Shapiro-Wilk test was conducted. The resulting *p*-values <0.0001 indicated that the data were not normally distributed. Due to the non-normal distribution, Spearman correlation analysis was used to calculate the correlation coefficient (*r*). Additionally, linear regression analysis was performed to determine the linear relationship between the two immobilization strategies (*Y* = *mX* + *b*). To compare paired measurements from the two immobilization strategies (DBCO- vs COOH-beads) the Wilcoxon signed-rank test (a paired, nonparametric test) was applied. Finally, to visualize the overall data distribution, Tukey’s method was used, as it is robust to skewed data and does not assume normality.

## Supplementary Material



## Notes

Figure S1: PEAKS assignment of peptides in human antigen sequences after LC-ESI-MS/MS analysis; Figure S2: SDS-PA gels after purification of human antigens; Figure S3: SDS-PA gels after fluorophore labeling of HDAC3 AzK; Figure S4: SDS-PA gel and immunodetection after RPS4Y1 AzK immobilization on DBCO-agarose beads; Figure S5: Plot of Luminex bead functionalization with DBCO groups; Figure S6: Plots of Luminex bead functionalization with COOH groups; Figure S7: Plots of immobilization of human antigens on DBCO-functionalized beads; Figure S8: Plot of immobilization of human antigens on COOH-functionalized beads; Figure S9: Bar charts showing antigen immobilization after blocking of DBCO-functionalized beads; Figure S10: Bar chart showing blocking of DBCO-functionalized beads in the absence of antigen; Figure S11: Hydropathy plots of human antigens; Figure S12: Cartoon 3D structures of human antigenic proteins with all primary amines highlighted; Figure S13: Plot of immobilization of RPS4Y1 AzK on DBCO- and COOH-functionalized beads, and Table S1: DNA and amino acid sequences (PDF)
