# Supplementary material for: Site-Specific Antigen Immobilization Improves Autoantibody Binding Efficiency on the Luminex Platform
Source: ACS Omega. 2026 Apr 10;11(15):22766–86. doi: 10.1021/acsomega.5c11987 (PMC13103837; doi:10.1021/acsomega.5c11987)
Supplement: Supplementary file 1 [file ao5c11987_si_001.pdf]

Supporting Information

Site-specific antigen immobilization improves autoantibody binding efficiency on the Luminex platform

Dajana Kolanovic[a,b], Manuela Hofner[c], Jasmin Huber[c], Andreas Weinhaeusel[c,*], Birgit Wiltschi[a,d,*]

[a] acib – Austrian Centre of Industrial Biotechnology, Petersgasse 14, 8010 Graz, Austria

[b] Institute of Molecular Biotechnology, Graz University of Technology, Petersgasse 14, 8010 Graz, Austria

[c] Molecular Diagnostics, AIT Austrian Institute of Technology GmbH, Giefinggasse 4, 1210 Vienna, Austria.

[d] Institute of Bioprocess Science and Engineering, Department of Biotechnology and Food Sciences, BOKU University, Muthgasse 18, 1190 Vienna, Austria

*Corresponding author.

E-mail address: birgit.wiltschi@acib.at (B. Wiltschi)

E-mail address: Andreas.Weinhaeusel@ait.ac.at (A. Weinhaeusel)

Supporting Figures

A

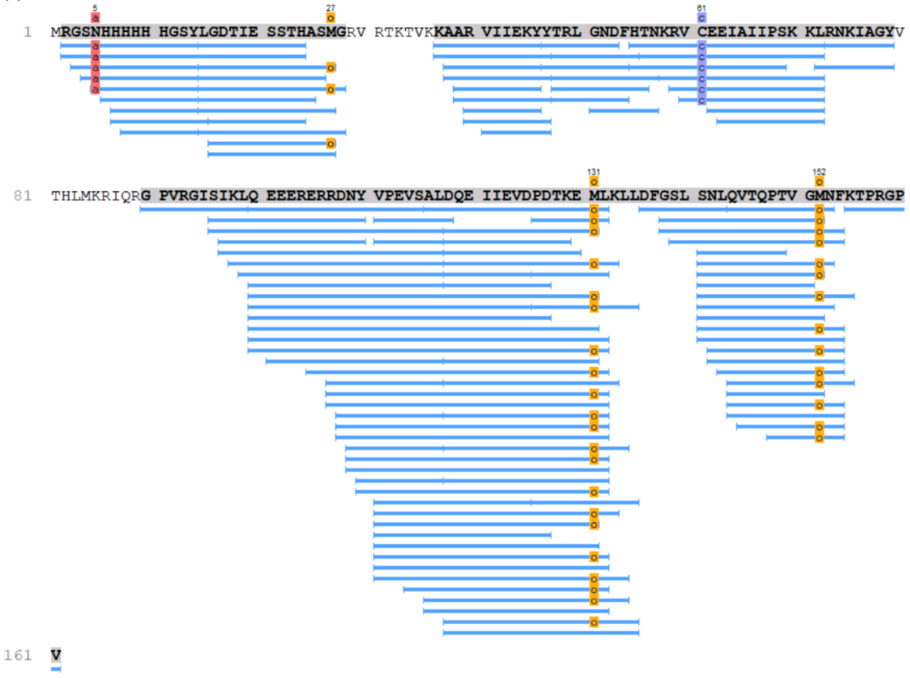

B

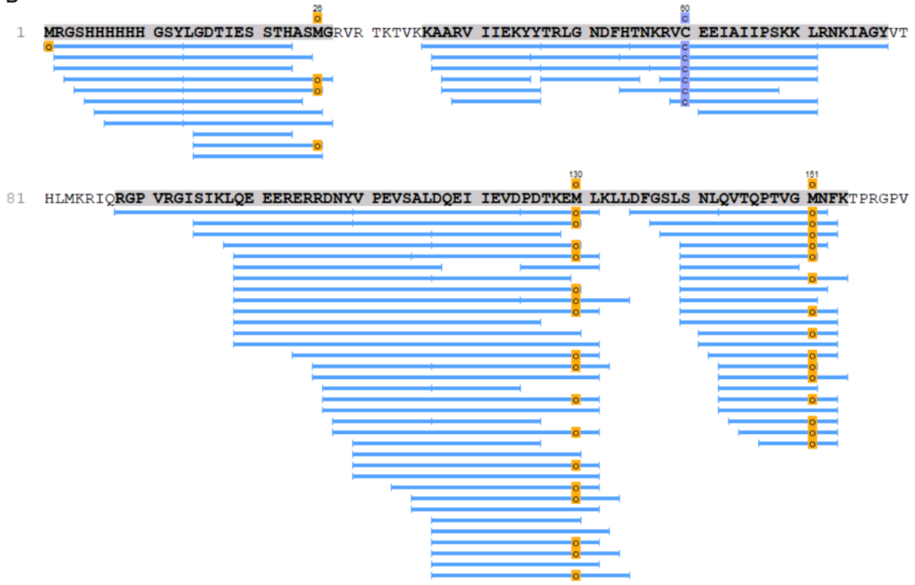

C

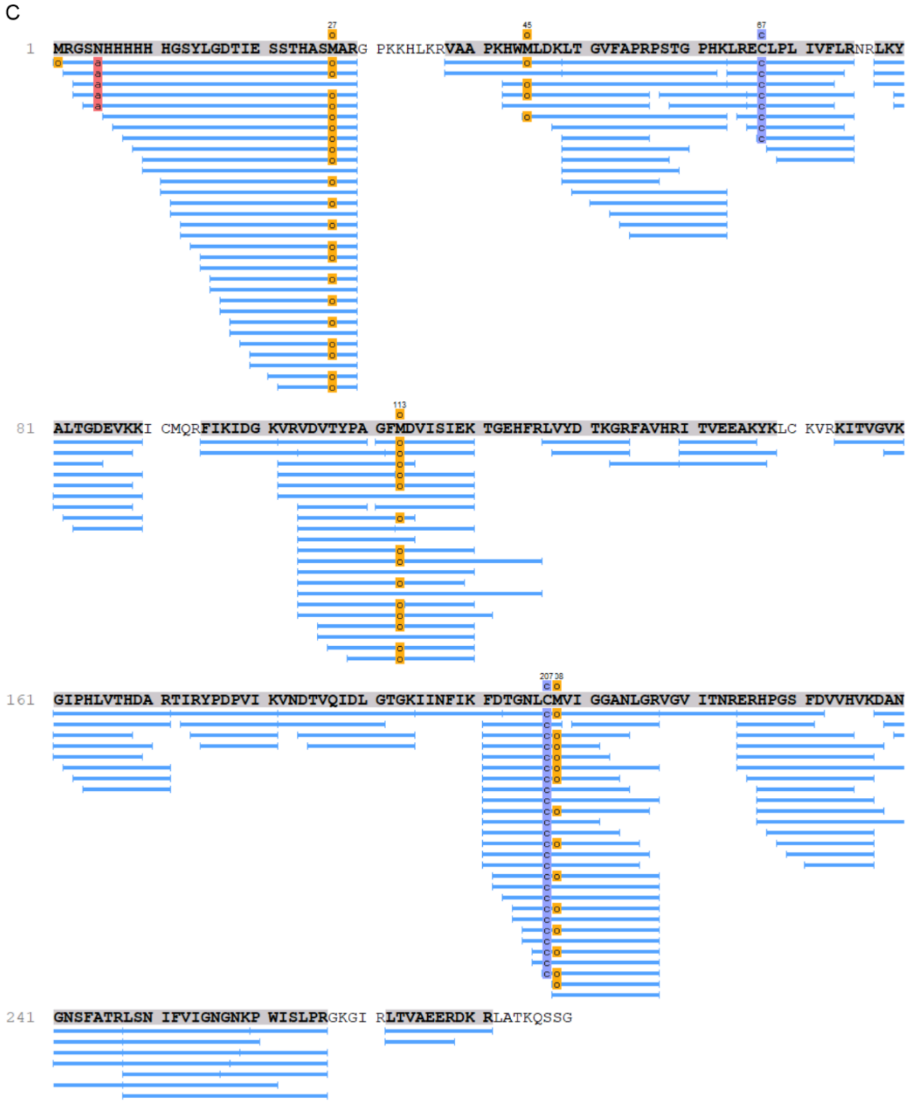
27
45
67
1 MRGSNHHHHH HGSYLGDTIE SSTHASMARG PKKHLKRVAA PKHWMLDKLT GVFAPRPSTG PHKLRECLPL IVFLRNRLKY
113
81 ALTGDEVKKI CMQRFIKIDG KVRVDVTYPA GFMDVISIEK TGEHFRLVYD TKGRFAVHRI TVEEAKYKLC KVRKITVGVK
207 38
161 GIPHLVTHDA RTIRYPDPVI KVNDTVQIDL GTGKIINFIK FDTGNLCMVI GGANLGRVGV ITNRERHPGS FDVVHVKDAN
241 GNSFATRLSN IFVIGNGNKP WISLPRGKGI RLTVAEERDK RLATKQSSG

D

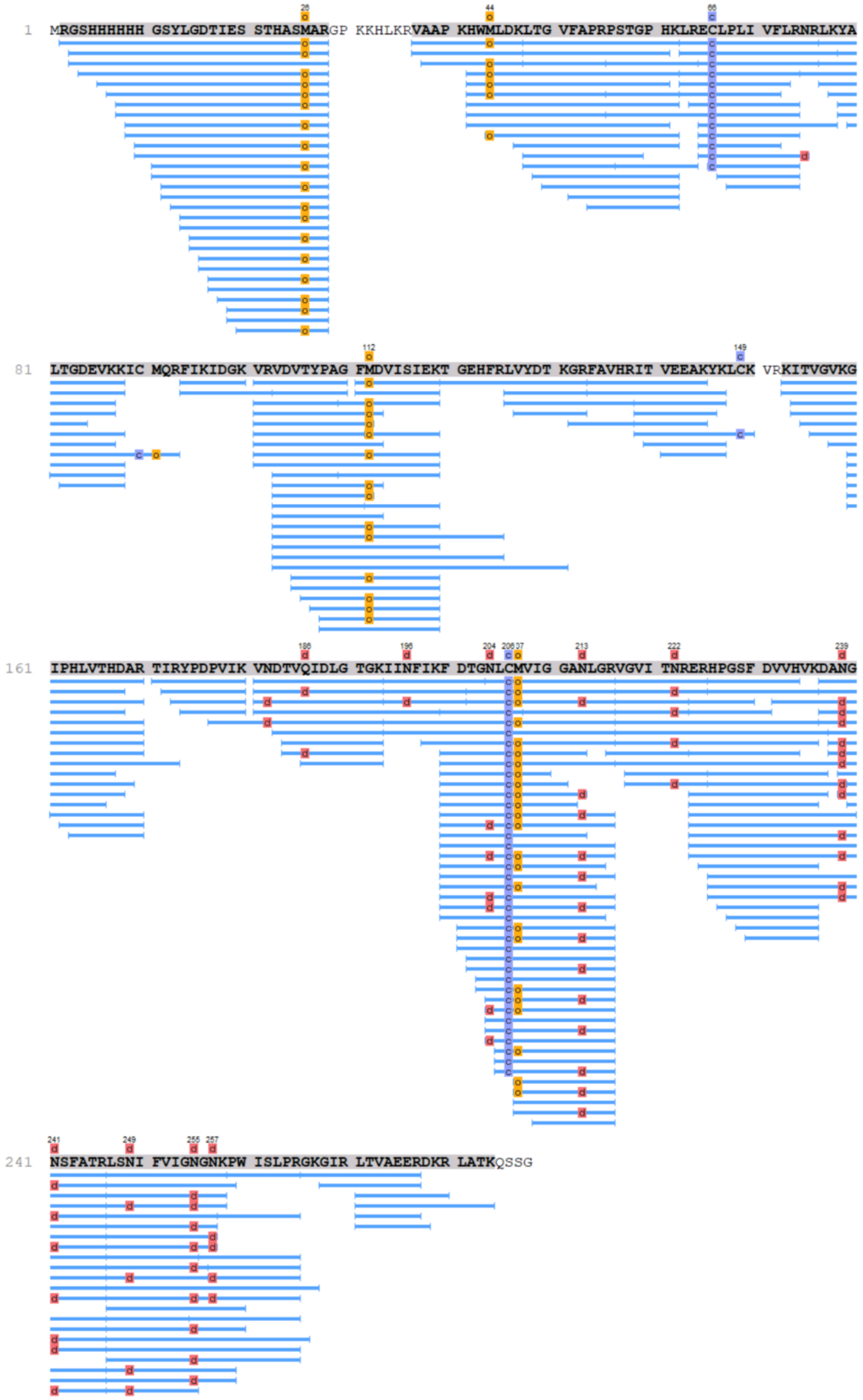

1 MRGSHHHHHH GSYLGDTIES STHASMARGP KKHLKRVAAP KHWMLDKLTG VFAPRPSTGP HKLRECLPLI VFLRNRLKYA
81 LTGDEVKKIC MQRFIKIDGK VRVDVTYPAG FMDVISIEKT GEHFRLVYDT KGRFAVHRIT VEEAKYKLCK VRKITVGVKG
161 IPHLVTHDAR TIRYPDPVIK VNDTVQIDLG TGKIINFIKF DTGNLCMVIG GANLGRVGVI TNRERHPGSF DVVHVKDANG
241 NSFATRLSNI FVIGNGNKPW ISLPRGKGIR LTVAEERDKR LATKQSSG

E

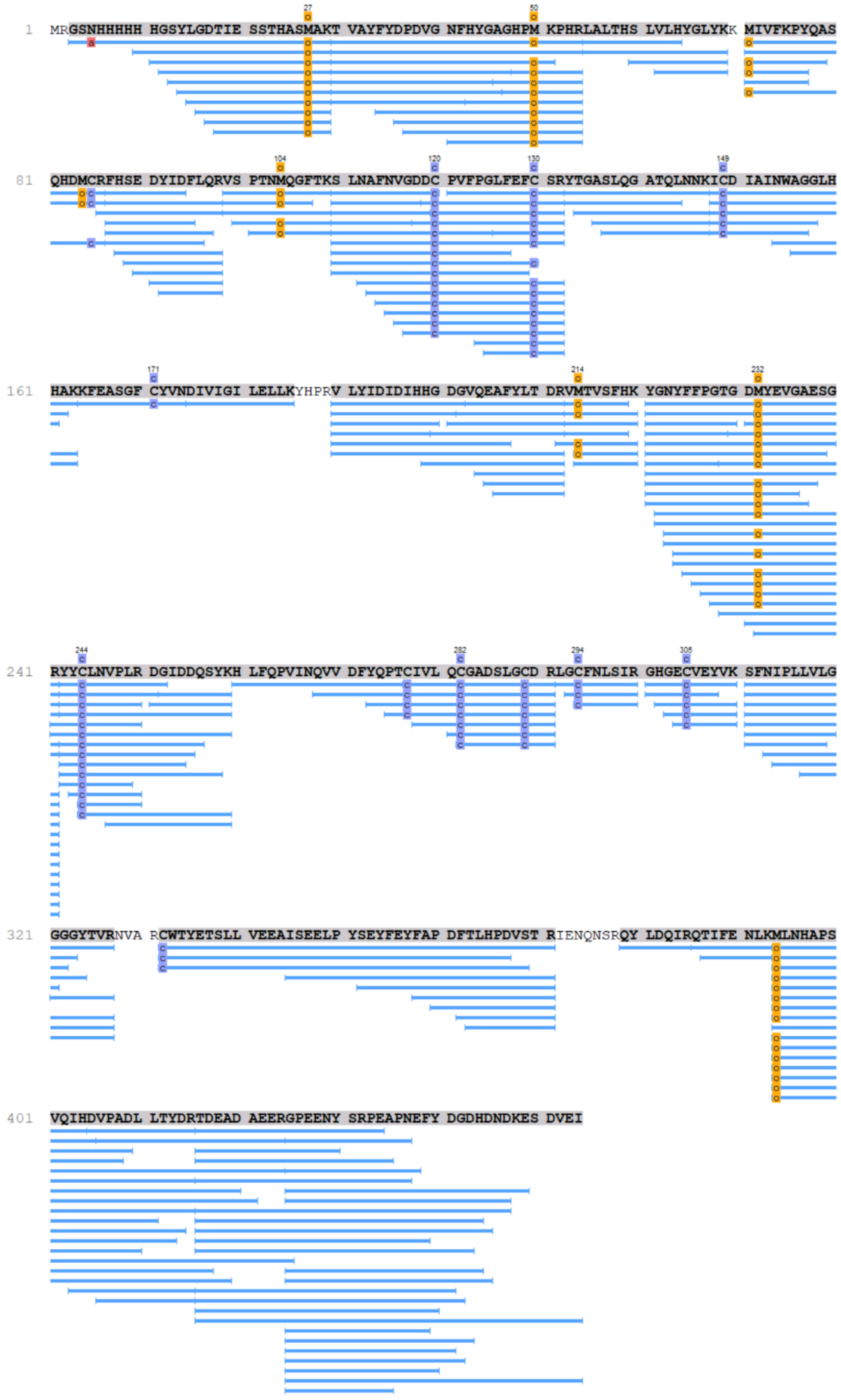

27
50
1 MRGSNHHHHH HGSYLGDTIE SSTHASMAKT VAYFYDPDVG NFHYGAGHPM KPHRLALTHS LVLHYGLYKK MIVFKPYQAS
104
120
130
149
81 QHDMCRFHSE DYIDFLQRVS PTNMQGFTKS LNAFNVGDDC PVFPGLFEFC SRYTGASLQG ATQLNNKICD IAINWAGGLH
171
214
232
161 HAKKFEASGF CYVNDIVIGI LELLKYHPRV LYIDIDIHHG DGVQEAFYLT DRVMTVSFHK YGNYFFPGTG DMYEVGAESG
244
282
294
305
241 RYYCLNVPLR DGIDDQSYKH LFQPVINQVV DFYQPTCIVL QCGADSLGCD RLGCFNLSIR GHGECVEYVK SFNIPLLVLG
321 GGGYTVRNVA RCWTYETSLL VEEAISEELP YSEYFEYFAP DFTLHPDVST RIENQNSRQY LDQIRQTIFE NLKMLNHAPS
401 VQIHDVPADL LTYDRTDEAD AEERGPEENY SRPEAPNEFY DGDHDNDKES DVEI

F

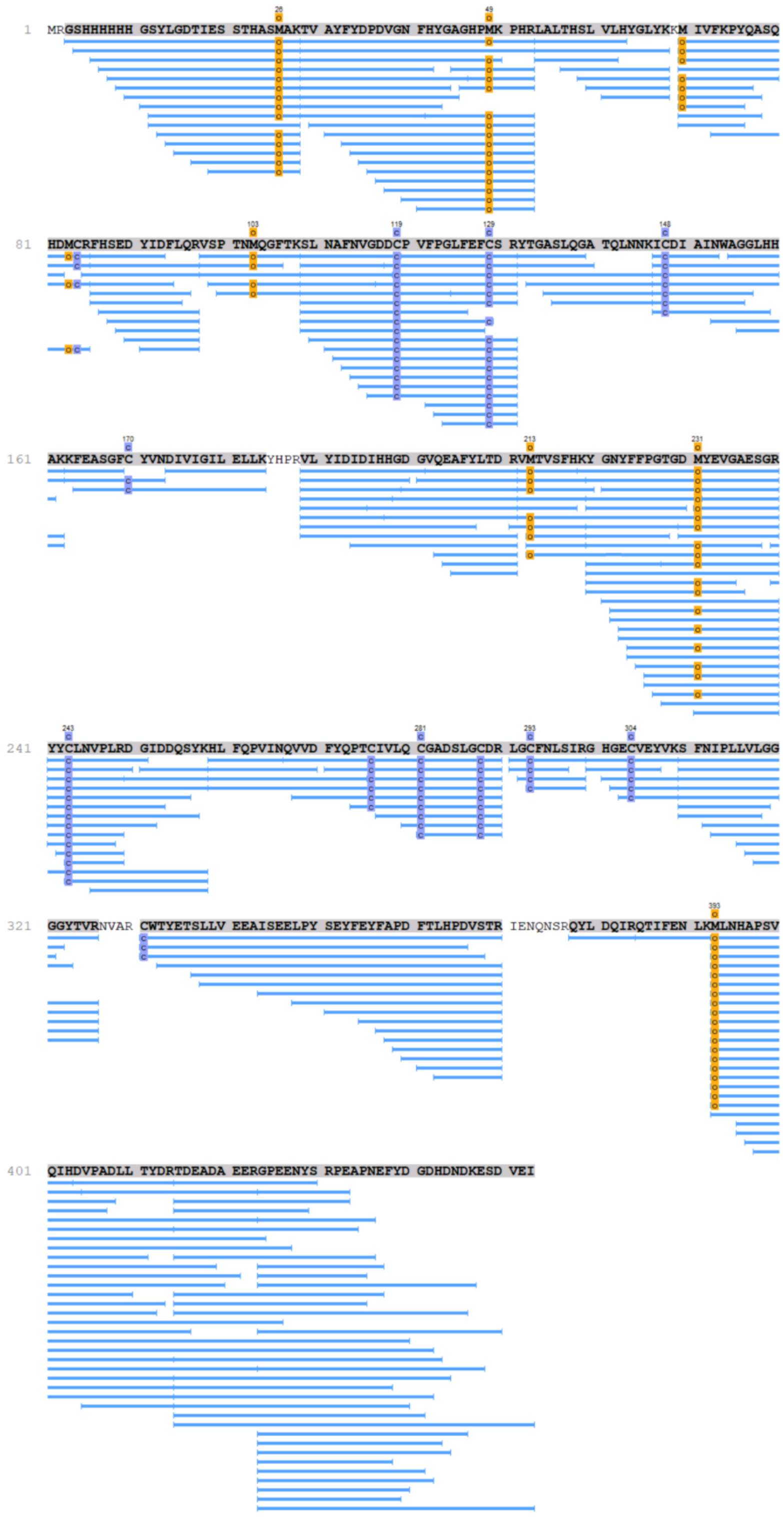

Figure S1 Mass spectrometry analysis confirmed protein identity and AzK incorporation into the different human antigens. (A) RPS17 AzK; (B) RPS17 wt; (C) RPS4Y1 AzK; (D) RPS4Y1 wt; (E) HDAC3 AzK; (F) HDAC3 wt. PEAKS software was used to assign peptides to the sequence of interest. Sequence stretches highlighted in gray indicate identified peptides. Each blue bar represents a detected

peptide sequence. Putative post-translational modifications (PTMs) and mutations are marked with colored squares: d, deamidation of asparagine or glutamine; o, oxidation of methionine; c, carbamidomethylation of cysteine. Highly confident PTMs and mutations are indicated on the top of the protein sequence. An asparagine (N) residue was introduced at position 5 of the protein sequence in place of AzK, as the software does not recognize ncAAs. A mass difference corresponding to AzK (+127.07 g/mol) was allowed at the N residue to account for its incorporation. The label 'a' denotes the successful identification of AzK at position 5.

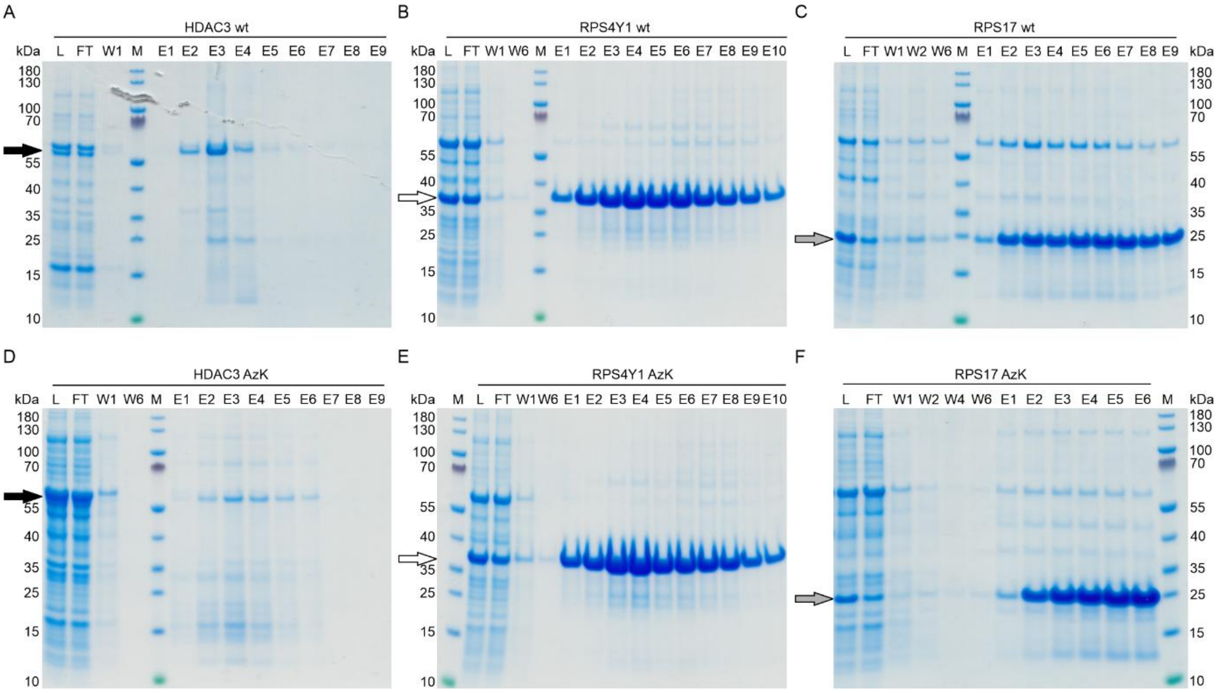

Figure S2 Purification of HDAC3 wt, RPS4Y1 wt, RPS17 wt and their AzK variants using Ni-IMAC. We solubilized inclusion bodies and purified hexahistidine-tagged proteins by nickel affinity chromatography. The purified proteins were analyzed on a 4-12% SDS-PA gel and the results are shown in panels (A) HDAC3 wt, (B) RPS4Y1 wt, (C) RPS17 wt, (D) HDAC3 AzK, (E) RPS4Y1 AzK, and (F) RPS17 AzK. The analysis indicated that these proteins were successfully purified by Ni-IMAC, while HDAC3 wt and its AzK variant were poorly expressed. In all purified samples, additional bands were observed, likely corresponding to co-purified *E. coli* host proteins. Lanes L, solubilized inclusion bodies; FT, flowthrough; W, column wash; E, eluate; M, molecular size marker. The numbers on the margins of the gels indicate the size of the molecular weight marker bands in kDa. The MW_{calc} of RPS17 wt and its AzK variant (gray arrow), RPS4Y1 wt and RPS4Y1 AzK (white arrow), and HDAC3 wt and HDAC3 AzK are indicated in the caption to Figure 2. The gels were stained with Coomassie protein stain.

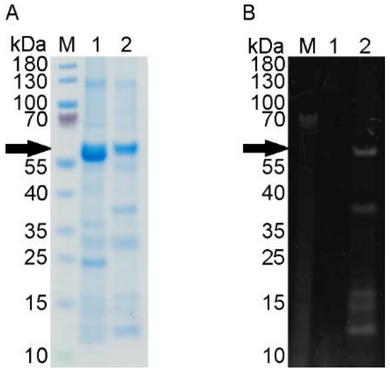

Figure S3 Confirmation of AzK incorporation in the HDAC3 azido-variant by fluorophore labeling via SPAAC. Following scale-up expression, IMAC-purified HDAC3 wt (lane 1) and HDAC2 AzK (lane 2) were incubated with DBCO-Cy3 fluorophore and analyzed by SDS-PAGE. (A) The proteins were stained with Coomassie stain. (B) The SDS-PA gel, exposed to UV light before Coomassie staining, shows a fluorescent band resulting from the incubation of AzK variant with DBCO-Cy3 (lane 2). This confirms the presence of the azide group in the HDAC3 AzK variant, which is required for conjugation with the alkyne-containing fluorophore. The negative control, HDAC3 wt (lane 1), which lacks the azide group for bioorthogonal conjugation, did not display a fluorescent band with the DBCO-Cy3, confirming the specificity of the conjugation reaction. Lane M, molecular size marker; lane 1, HDAC3 wt; lane 2, HDAC3 AzK. The numbers on the left margins of the gels indicate the size of the molecular weight marker bands in kDa. The black arrows indicate HDAC3 wt and its AzK variant (MW_{calc} ~ 52 kDa).

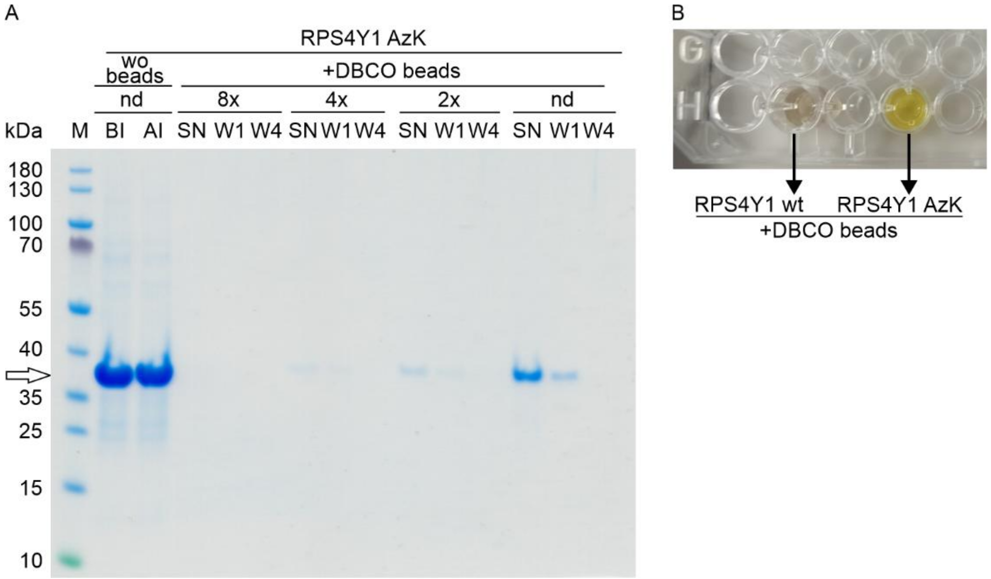

Figure S4 Validation of RPS4Y1 AzK immobilization on DBCO-agarose beads. (A) SDS-PAGE analysis of RPS4Y1 AzK incubated with or without DBCO-agarose beads. Proteins were incubated for 24 h, and supernatants (SN) and wash fractions (W1, W4) were analyzed. Without beads (wo beads), RPS4Y1 AzK remained in solution, indicating no loss due to degradation or instability. When incubated with DBCO-agarose beads, a portion of RPS4Y1 AzK was retained in the supernatant at standard concentration (nd, not diluted). However, upon diluting the protein sample 8-fold (8x), complete immobilization was achieved, as indicated by the absence of protein in the SN lane. Partial immobilization was observed at 4x and 2x dilutions. Lanes M, molecular size marker; BI, before incubation; AI, after incubation. The numbers on the left margin of the gel represent the size of the molecular weight marker bands in kDa. The white arrow indicates RPS4Y1 AzK (MW_{calc} = 32.5 kDa). (B) Immunodetection of RPS4Y1 AzK immobilization on DBCO-agarose beads. Beads were incubated with either RPS4Y1 AzK or RPS4Y1 wt (negative control), followed by detection using an anti-6H primary antibody and an HRP-conjugated secondary antibody. Addition of TMB substrate resulted in a yellow color in the RPS4Y1 AzK sample, confirming successful immobilization. No yellow color was observed for the RPS4Y1 wt sample, indicating the absence of nonspecific binding.

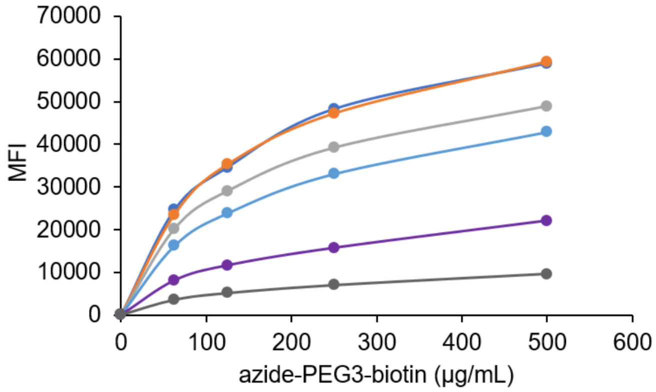

Figure S5 Optimization of Luminex bead functionalization with DBCO groups. Carboxylated MagPlex beads were functionalized with DBCO groups via amine coupling using sulfo DBCO-PEG4-amine linker at six different concentrations: 500 µg/mL (orange), 250 µg/mL (dark blue), 125 µg/mL (light gray), 62.5 µg/mL (light blue), 15.6 µg/mL (purple), 3.9 µg/mL (dark gray). Functionalization efficiency was assessed using SPAAC with azide-PEG3-biotin at final concentrations of 500 µg/mL, 250 µg/mL, 125 µg/mL, and 62.5 µg/mL. The incorporation of biotin enabled subsequent Streptavidin-R-Phycoerythrin (SA-PE) binding, measured as median fluorescence intensity (MFI). Results show that a sulfo DBCO-PEG4-amine concentration of 250 µg/mL was required to achieve saturation of DBCO groups on the beads, as increasing the linker concentration to 500 µg/mL did not further enhance MFI. Lower linker concentrations resulted in reduced functionalization efficiency. A control (blank) consisting of MES buffer without azide-PEG3-biotin (0 µg/mL) resulted in no fluorescence signal, confirming the specificity of SA-PE binding to the biotin moiety of azide-PEG3-biotin. Each azide-PEG3-biotin concentration, including the MES buffer control, was tested in duplicate. Data points represent the average MFI of two technical replicates.

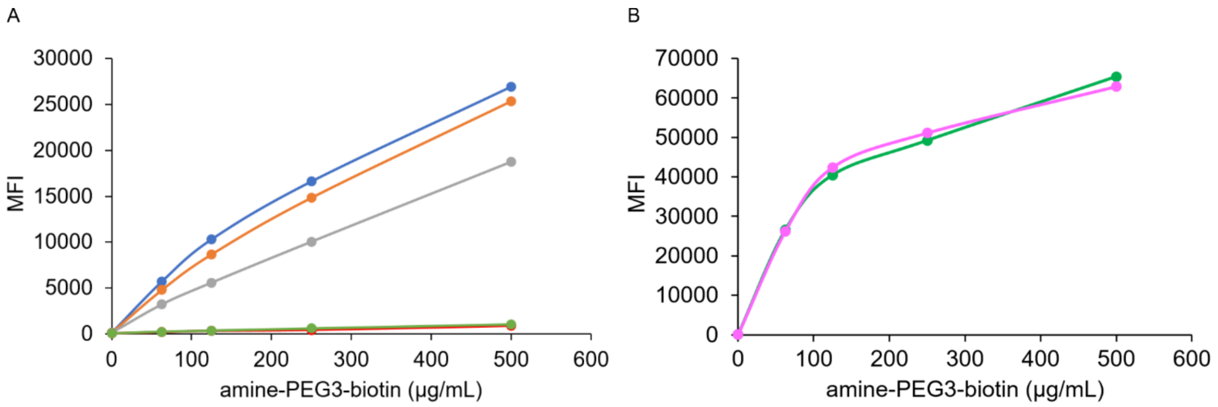

Figure S6 Luminex beads were successfully functionalized with COOH groups. (A) MagPlex beads were functionalized with COOH groups via amine coupling using amino-PEG4-COOH at 100 µg/mL (dark blue), 50 µg/mL (orange), and 25 µg/mL (light gray). Functionalization efficiency was assessed by subsequent amine coupling of amine-PEG3-biotin at 500, 250, 125, and 62.5 µg/mL, enabling SA-PE binding and fluorescence detection as MFI. Higher amino-PEG4-COOH concentrations improved functionalization efficiency; however, saturation of COOH groups was not achieved. A control (blank) using MES buffer without amine-PEG3-biotin (0 µg/mL) exhibited no fluorescence, confirming SA-PE binding specificity. To assess functionalization specificity, amino-PEG4-COOH and amine-PEG3-biotin were added without prior EDC/sulfo-NHS activation (red). This resulted in no fluorescence, which ruled out nonspecific binding of either linker to the beads. In a second control, beads were activated with EDC/sulfo-NHS, but amino-PEG4-COOH was omitted. After passivation with ethanolamine, amine-PEG3-biotin was added without additional EDC/sulfo-NHS activation (light green), producing no fluorescence. This confirmed that ethanolamine effectively quenched amine-reactive sulfo-NHS esters, thereby preventing unintended coupling. (B) MagPlex beads were functionalized with COOH groups using amino-PEG4-COOH at 250 µg/mL (dark green) and 500 µg/mL (pink). Saturation of COOH groups was achieved at 250 µg/mL, as increasing the linker concentration to 500 µg/mL did not enhance MFI, indicating no further functionalization. Each amine-

PEG3-biotin concentration, including the MES buffer control, was tested in duplicate. Data points represent the average MFI of two technical replicates.

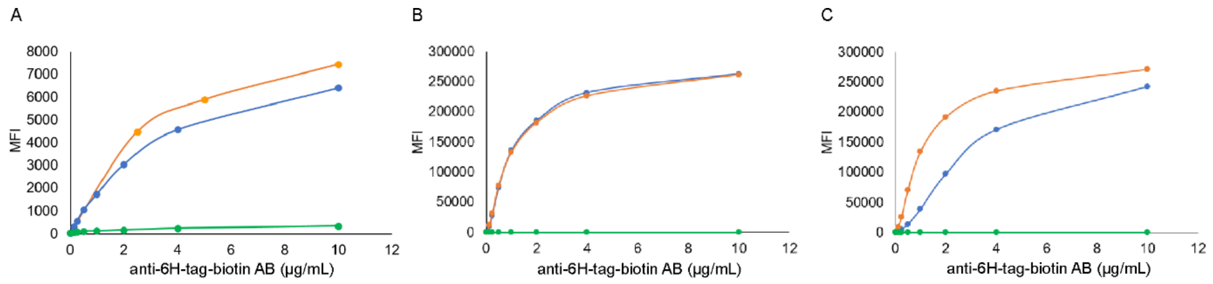

Figure S7 Human antigenic proteins exhibited nonspecific binding to DBCO-functionalized beads. AzK-functionalized antigens (blue) were conjugated to DBCO-functionalized beads via SPAAC. To assess nonspecific binding, wild-type antigens lacking the azide reactive group were used as a negative control (orange). Antigens were prepared in MES buffer, pH 5, and an additional control with MES buffer alone (green) was included to confirm assay specificity. Immobilization efficiency was evaluated using an anti-6H-tag-biotin antibody, which specifically recognizes proteins containing the 6H-tag, followed by SA-PE binding for fluorescence detection. The anti-6H-tag-biotin antibody was tested at final concentrations of 10, 4, 2, 1, 0.5, 0.25, and 0.13 µg/mL. A control (blank) consisting of assay buffer (PBS containing 1% (w/v) BSA, 0.05% (w/v) NaN_3, pH 7.4) without antibody (0 µg/mL) was also included. Each antibody concentration, including the assay buffer control, was tested in duplicate. Data points represent the average MFI of two technical replicates. Panels show results for (A) HDAC3, (B) RPS4Y1, and (C) RPS17. The data indicate successful immobilization of AzK-functionalized proteins on DBCO-functionalized beads, as demonstrated by elevated fluorescence signals (MFI). However, wt antigens also exhibited substantial binding to the beads, revealing nonspecific interactions. The minimal fluorescence signal from the MES buffer control confirmed that the anti-6H-tag-biotin antibody and detection reagents interacted only negligibly with the beads.

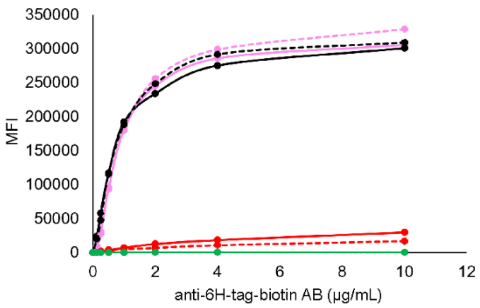

Figure S8 Human antigenic proteins exhibited nonspecific binding to COOH-functionalized beads. Wild-type antigens HDAC3 wt (red lines), RPS4Y1 wt (black lines), and RPS17 wt (pink lines) were conjugated to COOH-functionalized beads either with (solid lines) or without (dashed lines) EDC/sulfo-NHS activation. Antigens were prepared in MES buffer, pH 5, and MES buffer alone (green line) was included as a negative control to confirm assay specificity. Immobilization efficiency was assessed using an anti-6H-tag-biotin antibody, followed by SA-PE binding, and the resulting fluorescence signals were measured as MFI. The anti-6H-tag-biotin antibody was tested at final concentrations of 10, 4, 2, 1, 0.5, 0.25, and 0.13 µg/mL. A control (blank) consisting of assay buffer without antibody (0 µg/mL) was also included. Each anti-6H-tag-biotin antibody concentration, including the assay buffer control, was tested in duplicate. Data points represent the average MFI of two technical replicates. As expected, the results demonstrate successful immobilization of wt antigens on COOH-functionalized beads after EDC/sulfo-NHS activation (solid lines), as evidenced by increased MFI. However, substantial nonspecific binding of wt antigens to the beads was also observed in the absence of EDC/sulfo-NHS activation (dashed lines). The MES buffer control (green line) exhibited no increase in MFI, confirming that the anti-6H-tag-biotin antibody and detection reagents did not bind nonspecifically to the beads.

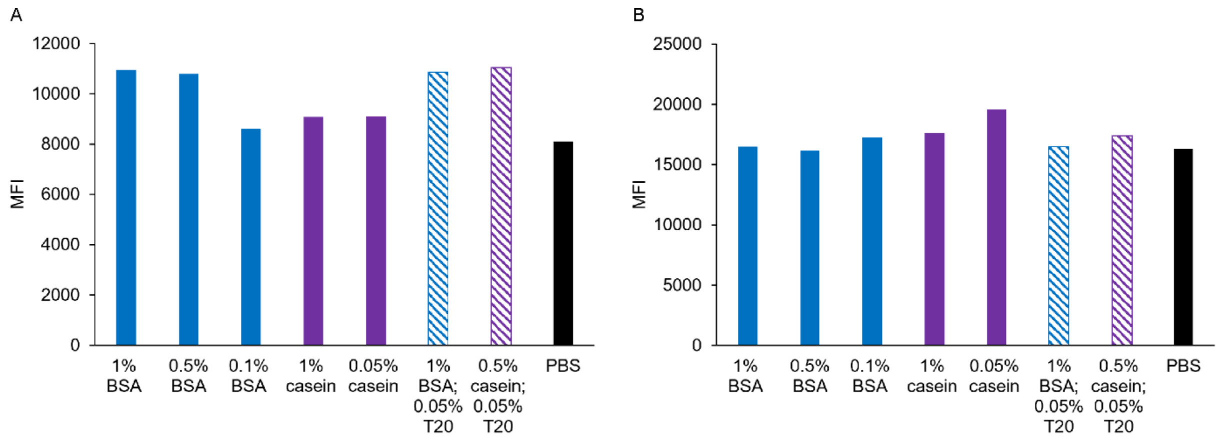

Figure S9 Blocking does not reduce nonspecific binding of HDAC3 and RPS17 to DBCO-functionalized beads. To minimize nonspecific antigen adsorption, DBCO-functionalized beads were blocked with various blocking agents before the addition of HDAC3 wt and RPS17 wt. Since HDAC3 wt and RPS17 wt lack an azido group, they cannot covalently react with DBCO, meaning any observed binding represents nonspecific interactions. Bovine serum albumin (BSA) (blue bars) and casein (purple bars) were tested at different concentrations, along with combinations containing 0.05% (v/v) Tween 20 (T20) (hatched bars). All blocking reagents were prepared in PBS, pH 7.4. PBS alone, which lacks blocking properties, served as the control (black bars). After incubation, antigen binding was detected using an anti-6H-tag-PE antibody at a final concentration of 10 µg/mL. Antibody detection was performed in duplicate, and MFI was measured. Bars represent the average MFI of two technical replicates. (A) MFI values of HDAC3 wt bound to DBCO-beads show that blocking did not reduce nonspecific binding, as evidenced by MFI values higher than those from the PBS control. (B) MFI values of RPS17 wt bound to DBCO-beads demonstrate that none of the tested blocking agents effectively reduced nonspecific binding. To confirm that fluorescence signals originated from specific antigen interactions rather than nonspecific interactions between blocking and detection reagents, fluorescence measurements were taken from blocked beads in the absence of antigen (Figure S10).

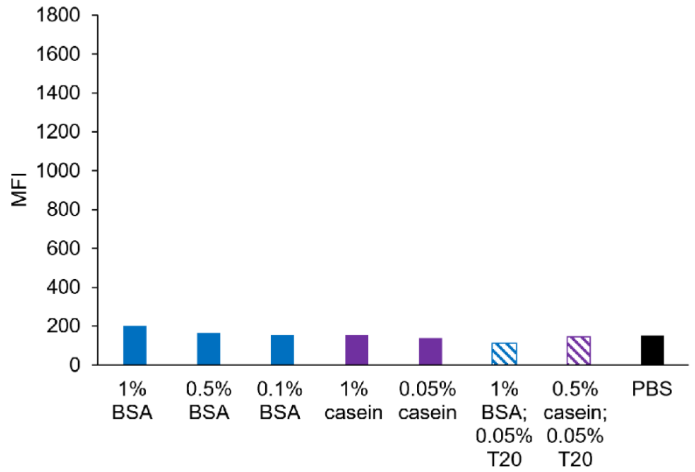

Figure S10 Fluorescence measurement after blocking DBCO-functionalized beads in the absence of antigen. DBCO-beads were blocked with various concentrations of bovine serum albumin (BSA) (blue bars) and casein (purple bars), with some conditions also containing 0.05% (v/v) Tween 20 (T20) (hatched bars). Unblocked beads incubated in PBS alone (black bar) served as a reference. After incubation, only elution buffer (without antigen) was added, and fluorescence was measured using the anti-6H-tag-PE antibody at a final concentration of 10 µg/mL. Antibody detection was performed in duplicate, and bars represent the average MFI of two technical replicates. The low fluorescence (MFI ~200) from unblocked beads without antigen indicates minimal binding of the detection antibody to the beads. Similarly, consistently low MFI values across all blocking conditions without antigen, confirm that there is no substantial nonspecific binding of the anti-6H-tag-PE antibody to the blocking reagents. These results demonstrate that the fluorescence signals detected in Figure 8 and Figure S9 resulted from antigen interactions with the anti-6H-tag-PE antibody rather than from nonspecific binding of the detection antibody to blocking reagents.

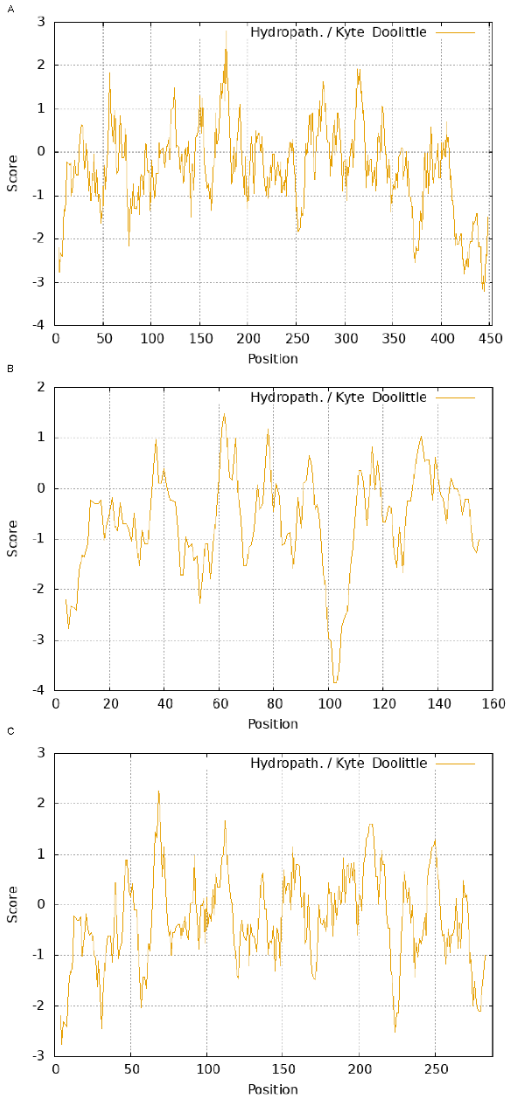

Figure S11 Hydropathy analysis of human antigenic proteins. Hydropathy plots of (A) HDAC3 wt, (B) RPS17 wt, and (C) RPS4Y1 wt were generated using the Kyte-Doolittle scale via the ExPASy ProtScale tool (https://web.expasy.org/protscale/). The plots display the distribution of hydrophobic and hydrophilic regions along the amino acid sequence of each protein. Positive values indicate hydrophobic regions, while negative values represent hydrophilic regions. HDAC3 wt shows extended hydrophobic stretches (scores ≥ +2), RPS17 wt presents an overall hydrophilic profile punctuated by localized hydrophobic peaks, and RPS4Y1 wt exhibits a more balanced profile with alternating regions and minimal extended hydrophobic stretches. These patterns support observed variations in solubilization efficiency and nonspecific binding behavior under denaturing conditions.

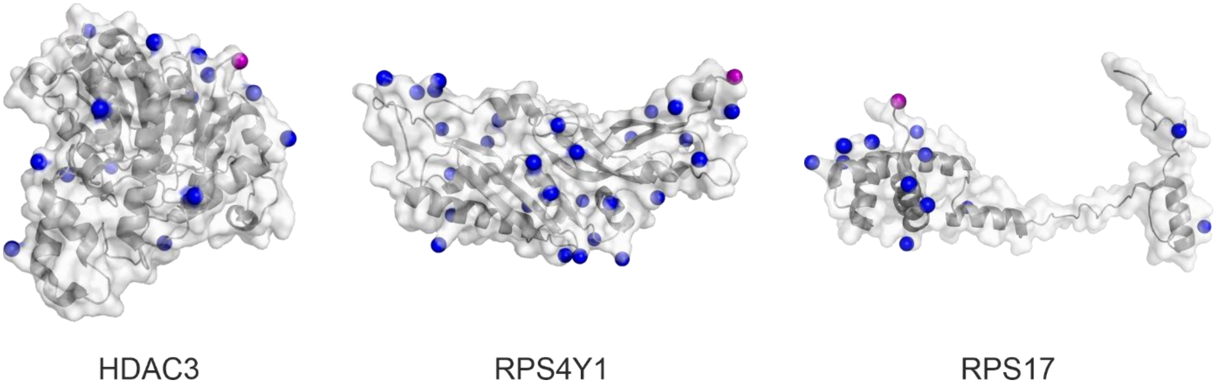

Figure S12 Exposure of primary amines on the surface of the antigen proteins. Protein structures (HDAC3, PDB 4A69; RPS4Y1, PDB 6OLG (chain BB [auth BE]); RPS17, PDB 8GLP (chain Z [auth SR])) are shown as cartoons (grey) with the surrounding surface (transparent). Primary amines of lysine residues are shown as blue spheres, the N-terminal primary amine is shown in purple.

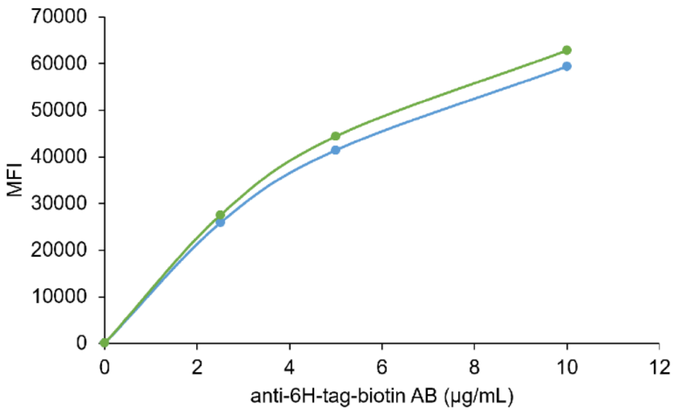

Figure S13 Confirmation of RPS4Y1 AzK immobilization on DBCO- and COOH-beads. The plot shows signal readings of RPS4Y1 AzK after coupling to DBCO-beads (blue) and COOH-beads (green). Coupling was confirmed by the binding of an anti-6H-tag-biotin antibody to the immobilized antigen, followed by detection with SA-PE. The anti-6H-tag-biotin antibody was tested at final concentrations of 10, 5, and 2.5 µg/mL. A control (blank) consisting of assay buffer without antibody (0 µg/mL) was also included. Each anti-6H-tag-biotin antibody concentration, including the assay buffer control, was tested in duplicate. Data points represent the average MFI of two technical replicates. The comparable signal intensities for both immobilization strategies suggest similar antigen loading levels on DBCO- and COOH-beads.

Supporting Tables

Table S1 DNA and amino acid sequences used in this study.

Name	Type	DNA sequence (5' → 3')
RPS17	gene fragment[a], [b]	CATATGAGAGGATCG*CATCACCATCACCATCACGGATCCTATT<br>TAGGTGACACTATAGAATCGTCGACCCACGCGTCCATGGGTCGG<br>GTACGGACCAAGACCGTGAAAAAAGCTGCGCGGGTTATTATAGA<br>GAAATATTATACACGGCTGGGTAACGATTTTCATACAAACAAAC<br>GGGTGTGTGAAGAAATTGCGATCATTCCGTCGAAGAAATTACGG<br>AACAAAATAGCAGGGTACGTTACACACCTGATGAAACGGATCCA<br>GCGTGGTCCAGTACGTGGGATTAGCATTAAACTGCAGGAAGAAG<br>AACGTGAGCGTCGTGATAATTACGTACCAGAAGTTTCCGCGTTA<br>GACCAGGAAATCATCGAGGTAGACCCAGATACGAAAGAGATGCT<br>GAAATTATTAGATTTTGGTTCACTGAGCAACTTACAGGTTACGC<br>AGCCAACGGTAGGTATGAATTTTAAGACACCACGGGGTCCAGTA<br>TAAAGATCT
RPS4Y1	gene fragment[a], [b]	CATATGAGAGGATCG*CATCACCATCACCATCACGGATCCTATT<br>TAGGTGACACTATAGAATCGTCGACCCACGCGTCCATGGCGCGG<br>GGTCCGAAAAAACATCTAAAACGAGTAGCAGCTCCCAAACACTG<br>GATGTTAGATAAGCTTACAGGGGTATTCGCACCACGACCATCAA<br>CGGGGCCGCATAAACTGCGTGAGTGCTTACCACTGATTGTTTTT<br>TTACGTAATCGTTTAAAATACGCTTTAACGGGTGACGAAGTAAA<br>AAAAATATGCATGCAACGATTTATTAAGATCGACGGTAAAGTAC<br>GGGTGGACGTTACGTATCCAGCCGGTTTTATGGACGTTATTTCG<br>ATTGAAAAAACGGGGGAGCACTTTCGGCTGGTTTACGATACCAA<br>AGGTCGATTCGCCGTACATCGGATTACGGTGGAGGAAGCAAAAT<br>ATAAATTATGTAAGGTGCGTAAAATCACGGTGGGTGTGAAAGGT<br>ATTCCACATCTGGTGACGCACGACGCCCGGACCATTCGGTATCC<br>AGACCCAGTTATTAAAGTGAACGACACGGTGCAGATCGACCTAG<br>GTACGGGTAAAATAATTAACTTCATTAAGTTCGACACGGGTAAT<br>TTATGCATGGTGATCGGGGGTGCGAACTTAGGGCGAGTAGGGGT<br>GATTACCAACCGTGAGCGTCACCCAGGGAGCTTCGACGTGGTGC<br>ACGTGAAAGACGCGAATGGTAACTCGTTCGCGACACGTTTGAGC<br>AACATCTTCGTTATCGGTAATGGTAATAAGCCATGGATCAGCCT<br>GCCGCGTGGTAAAGGTATCCGGTTAACGGTAGCCGAGGAACGTG<br>ACAAACGTCTGGCGACCAAGCAGTCGTCAGGTTAAAGATCT
HDAC3	gene fragment[a], [b]	CATATGAGAGGATCG*CATCACCATCACCATCACGGATCCTATT<br>TAGGTGACACTATAGAATCGTCGACCCACGCGTCCATGGCGAAA<br>ACCGTGGCGTACTTTTATGATCCGGATGTGGGTAACTTTCATTA<br>TGGTGCCGGTCATCCAATGAAACCGCACCGGCTGGCATTAACCC<br>ACTCGCTGGTTCTGCACTATGGGTTATACAAAAAAATGATTGTT<br>TTTAAACCATATCAGGCGAGCCAACACGATATGTGTCGGTTTCA<br>TAGCGAAGATTATATCGATTTTCTGCAGCGTGTTTCGCCGACCA<br>ATATGCAAGGTTTTACCAAATCATTAAATGCGTTTAACGTAGGT<br>GACGATTGTCCAGTGTTCCCGGGTTTATTCGAATTTTGTTCACG<br>ATATACGGGTGCAAGCCTGCAAGGTGCAACCCAGCTGAACAACA<br>AAATTTGCGACATCGCGATCAACTGGGCCGGGGGGCTGCATCAC<br>GCGAAAAAATTCGAAGCGAGCGGTTTTTGTTACGTTAACGATAT<br>CGTGATCGGTATTCTGGAACTGTTAAAATATCATCCACGGGTGT<br>TATATATCGATATCGATATTCATCACGGGGATGGTGTACAAGAG<br>GCCTTTTATTTAACGGATCGGGTTATGACAGTGAGCTTTCATAA<br>GTATGGTAATTATTTTTTTCCAGGTACGGGGGATATGTACGAGG<br>TTGGTGCAGAATCAGGTCGGTATTATTGCCTGAACGTGCCGCTG<br>CGGGACGGTATCGACGATCAGTCATATAAACATTTATTTCAGCC<br>CGTAATTAACCAGGTAGTGGATTTTTATCAACCGACATGTATCG<br>TGTTACAGTGCGGTGCCGATAGCCTGGGTTGCGACCGGTTAGGT<br>TGTTTCAACTTATCGATTCGGGGTCACGGTGAGTGTGTAGAGTA

		CGTTAAATCGTTTAATATTCCACTTTTAGTGCTGGGCGGCGGTG<br>GCTACACGGTTCGGAATGTAGCGCGGTGTTGGACGTACGAAACG<br>TCACTGCTGGTAGAGGAAGCGATCTCAGAAGAATTACCGTACTC<br>AGAGTATTTTGAATATTTCGCGCCAGATTTTACGTTACACCCAG<br>ACGTTTCGACCCGGATTGAAAATCAGAACTCCCGGCAGTACCTG<br>GATCAGATTCGGCAGACGATTTTCGAGAACCTGAAAATGCTGAA<br>CCACGCACCATCAGTTCAGATCCACGATGTGCCAGCAGATTTAC<br>TGACCTACGATCGTACGGACGAAGCCGACGCAGAAGAACGTGGG<br>CCAGAAGAAAACTACTCGCGTCCAGAAGCACCGAATGAATTTTA<br>CGACGGTGATCACGATAATGATAAAGAGTCGGACGTGGAAATCT<br>AAAGATCT

Name	Type	Amino acids sequence (N-terminus → C-terminus)
RPS17	protein[c], [d], [e]	MRGSXHHHHHHGSYLGDTIESSTHASMGRVRTKTVKKAARVIIE<br>KYYTRLGNDFHTNKRVCEEIAIIPSKKLRNKIAGYVTHLMKRIQ<br>RGPVRGISIKLQEEERERRDNYVPEVSALDQEIIEVDPDTKEML<br>KLLDFGSLSNLQVTQPTVGMNFKTPRGPV
RPS4Y1	protein[c], [d], [e]	MRGSXHHHHHHGSYLGDTIESSTHASMARGPKKHLKRVAAPKHW<br>MLDKLTGVFAPRPSTGPHKLRECLPLIVFLRNRLKYALTGDEVK<br>KICMQRFIKIDGKVRVDVTYPAGFMDVISIEKTGEHFRLVYDTK<br>GRFAVHRITVEEAKYKLCKVRKITVGVKGIPHLVTHDARTIRYP<br>DPVIKVNDTVQIDLGTGKIINFIKFDTGNLCMVIGGANLGRVGV<br>ITNRERHPGSFDVVHVKDANGNSFATRLSNIFVIGNGNKPWISL<br>PRGKGIRLTVAEERDKRLATKQSSG
HDAC3	protein[c], [d], [e]	MRGSXHHHHHHGSYLGDTIESSTHASMAKTVAYFYDPDVGNFHY<br>GAGHPMKPHRLALTHSLVLHYGLYKKMIVFKPYQASQHDMCRFH<br>SEDYIDFLQRVSPTNMQGFTKSLNAFNVGDDCPVFPGLFEFCSR<br>YTGASLQGATQLNNKICDIAINWAGGLHHAKKFEASGFCYVNDI<br>VIGILELLKYHPRVLYIDIDIHHGDGVQEAFYLTDRVMTVSFHK<br>YGNYFFPGTGDMYEVGAESGRYYCLNVPLRDGIDDQSYKHLFQP<br>VINQVVDFYQPTCIVLQCGADSLGCDRLGCFNLSIRGHGECVEY<br>VKSFNIPLLVLGGGGYTVRNVARCWTYETSLLVEEAISEELPYS<br>EYFEYFAPDFTLHPDVSTRIENQNSRQYLDQIRQTIFENLKMLN<br>HAPSVQIHDVPADLLTYDRTDEADAEERGPEENYSRPEAPNEFY<br>DGDHDNDKESDVEI

[a] Restriction sites are underlined.

[b] The asterisk (*) highlighted in pink marks the position where the amber stop codon (TAG) was introduced for site-specific incorporation of AzK.

[c] The linker MRGS, added at the N-terminus, is underlined.

[d] The introduced hexahistidine-tag is depicted in green, followed by the linker-adapter sequence (GSYLGDTIESSTHAS), shown in purple.

[e] The red X at position 5 indicates the site for AzK incorporation in human antigenic proteins.
